# Epidemiology and burden of progressive familial intrahepatic cholestasis: a systematic review

**DOI:** 10.1186/s13023-021-01884-4

**Published:** 2021-06-03

**Authors:** Tracey Jones-Hughes, Jo Campbell, Louise Crathorne

**Affiliations:** Roboleo & Co, Leeds, UK

**Keywords:** Pruritus, Bile acid, *ATP8B1*, *ABCB11*, *ABCB4*, *TJP2*, *NR1H4* and *Myo5b*

## Abstract

**Background:**

Progressive familial intrahepatic cholestasis is a rare, heterogeneous group of liver disorders of autosomal recessive inheritance, characterised by an early onset of cholestasis with pruritus and malabsorption, which rapidly progresses, eventually culminating in liver failure. For children and their parents, PFIC is an extremely distressing disease. Significant pruritus can lead to severe cutaneous mutilation and may affect many activities of daily living through loss of sleep, irritability, poor attention, and impaired school performance.

**Methods:**

Databases including MEDLINE and Embase were searched for publications on PFIC prevalence, incidence or natural history, and the economic burden or health-related quality of life of patients with PFIC. Preferred Reporting Items for Systematic Reviews and Meta-Analyses guidelines were followed.

**Results:**

Three systematic reviews and twenty-two studies were eligible for inclusion for the epidemiology of PFIC including a total of 2603 patients. Study periods ranged from 3 to 33 years. Local population prevalence of PFIC was reported in three studies, ranging from 9.0 to 12.0% of children admitted with cholestasis, acute liver failure, or splenomegaly. The most detailed data come from the NAPPED study where native liver survival of >15 years is predicted in PFIC2 patients with a serum bile  acid concentration below 102 µmol/L following bile diversion surgery. Burden of disease was mainly reported through health-related quality of life (HRQL), rates of surgery and survival. Rates of biliary diversion and liver transplant varied widely depending on study period, sample size and PFIC type, with many patients have multiple surgeries and progressing to liver transplant. This renders data unsuitable for comparison.

**Conclusion:**

Using robust and transparent methods, this systematic review summarises our current knowledge of PFIC. The epidemiological overview is highly mixed and dependent on presentation and PFIC subtype. Only two studies reported HRQL and mortality results were variable across different subtypes. Lack of data and extensive heterogeneity severely limit understanding across this disease area, particularly variation around and within subtypes.

**Supplementary Information:**

The online version contains supplementary material available at 10.1186/s13023-021-01884-4.

## Background

Progressive familial intrahepatic cholestasis (PFIC) is a rare, heterogeneous group of liver disorders of autosomal recessive inheritance, characterised by an early onset of cholestasis (usually during infancy) with pruritus and malabsorption, which rapidly progresses, eventually culminating in liver failure [1]. PFIC has a devastating impact on children’s lives, as well as on their parents and families. Unfortunately, without surgery or liver transplant (LT), only 50% of patients with PFIC survive up to the age of 10 years old and almost none to 20 years old [2].

PFIC is subgrouped according to the genetic defect, clinical presentation, laboratory findings, and liver histology (PFIC1 to PFIC6) [1]. The major mutations are in the *ATP8B1, ABCB11, ABCB4, TJP2, NR1H4*, or *Myo5b* genes, although some patients with PFIC have no identified mutation.

Benign recurring intrahepatic cholestasis (BRIC) is an episodic form of cholestasis. In practice, there is likely to be a spectrum of disease including PFIC and BRIC with patients having intermediate levels of cholestasis and long-term complications.

All types of PFIC are caused by defects in bile secretion from hepatocyte to canaliculi, however, distinct features are observed for each PFIC subtype, and the severity of the disease varies widely, including early onset-cirrhosis and hepatic cell carcinoma [1, 3]. Patients with the most common forms of PFIC (PFIC1 and PFIC2) generally present with jaundice and severe pruritus in the first few months of life.

For children and their parents, PFIC is an extremely distressing disease with pruritus being a key concern. Indeed, significant pruritus can lead to severe cutaneous mutilation (often drawing blood) and may affect many activities of daily living through loss of sleep, irritability, poor attention, and impaired school performance [4].

Regarding treatment, nutritional management is the initial step, where the patient’s formula is changed to a specialised one to maintain growth and manage malabsorption. The focus of pharmacological treatment is to relieve pruritus which is the most distressing symptom in PFIC. Other aims are to slow the disease progression, to improve the nutritional status, to correct vitamin deficiencies, and to treat the complications of advanced liver disease such as ascites and variceal bleeding. Not only are there no pharmaceutical treatments approved for use in this condition, but currently administered pharmacological options, such as ursodeoxycholic acid (UDCA) are minimally effective for PFIC. Therefore, the default treatment option is for physicians to exhaust all pharmaceutical options (including up to maximal dose) to the point where intractable pruritus, growth failure and nutritional deficiencies necessitate surgery or liver transplant.

Options for surgery include a number of techniques for biliary diversion (BD), which is used to interrupt the enterohepatic circulation of bile acids by diverting bile from the gallbladder, thereby decreasing the influx of bile acids to the gut and reuptake of bile acids in the small intestine and thereby lowering the bile acid pool. However, when BD fails to relieve symptoms or end-stage liver disease (ESLD) develops, LT is considered. Development of hepatocellular carcinoma (HCC) can also result in PFIC patients requiring a transplant.

## Aims

The aim of this review was to answer two research questions:

- What is the current evidence for the epidemiology and natural history of PIFC and BRIC?
- What is the current evidence for human and economic burden of PFIC and BRIC?

## Methods

### Literature search

During scoping, one systematic review (Baker et al., 2019) was identified which evaluated the epidemiology, natural history, and burden of PFIC [5]. This review was quality assessed and judged of moderate to high quality using AMSTAR. Based on initial scoping searches and pilot screening it was assumed that this review had sufficiently captured burden evidence for PFIC published before 2015.

The searches were customized to each of the databases were searched:

- MEDLINE (all) via Ovid SP
- Embase (1980 to present) via Ovid SP
- Cochrane Library (including: The Cochrane Database of Systematic Reviews (Cochrane Reviews); and The Cochrane Central Register of Controlled Trials (CENTRAL)
- CRD database including: Database of Abstracts of Reviews of Effects (DARE), Health Technology Assessment (HTA) Database; and The NHS Economic Evaluation Database (NHS EED)
- CRD International prospective register for systematic reviews (PROSPERO).

Bibliographies of SLRs were searched for eligible studies.

Conference proceedings for key conferences (annual proceedings) and HTA agency websites with English-language HTAs were also scrutinised.

Due to the rapidly evolving field of this rare disease, update searches were also conducted to ensure emerging data were captured.

### Study selection

The population of interest was people with PFIC or BRIC (adults or children). Studies had to be of an epidemiologic or natural history study design, with included evidence published in full text publications prior to or including 2015, or in abstracts prior to or including 2018. Studies were included if they met these pre-defined criteria.

Studies were excluded if their population was animals or in vitro, or if evidence was in the form of randomised controlled trials, editorials, letters, case reports, comments and non-systematic reviews. Studies were also excluded if they did not report on the pre-stated outcomes of interest, which are shown in Table 1.

**Table 1:** Outcomes of interest

Epidemiologic outcomes	Burden outcomes
Prevalence (any timeframe)	Generic quality of life scores measured via the following tools: EQ-5D HUI-1, HUI-2/HUI-3 SF6/SF6D/SF12, SF36 MOS, RAND12 or RAND 36 PedsQL
Incidence (any timeframe)	Utility values derived from generic preference-based instruments listed above
Disease natural history—disease progression	Disease-specific quality of life scores measures (any)
Mortality	Mapping studies, from disease-specific to generic preference-based measures or between different generic preference-based measures
	Disutilities associated with adverse events e.g. pruritus
	Caregiver utility values
	Descriptive summary of health states, and/or change in health status/QoL results

Titles, abstracts, and proceedings identified by the searches were screened using these pre-defined inclusion/exclusion criteria. Citations identified as eligible during title/abstract review were retrieved in full text for further review and screened in the same way. For full-text screening, excluded articles were documented with reasons for their exclusion according to the pre-defined criteria.

At each phase of the study selection process, the results were screened by two researchers and reconciled. A third researcher was consulted to reconcile any discrepancies.

The update searches were screened at title and abstract level and then full texts reviewed for inclusion. Included full texts were then compared to studies included in the original review to check for duplication.

Data extraction was split between two reviewers for included full text using a standardised data specification form, checked independently by another reviewer. Included full text studies were assessed by one researcher using the Joanna Briggs Institute (JBI) critical appraisal tool for prevalence studies [6]. Due to lack of detail, abstracts were not critically appraised.

## Results

### Research question 1: epidemiology

**Fig. 1:**
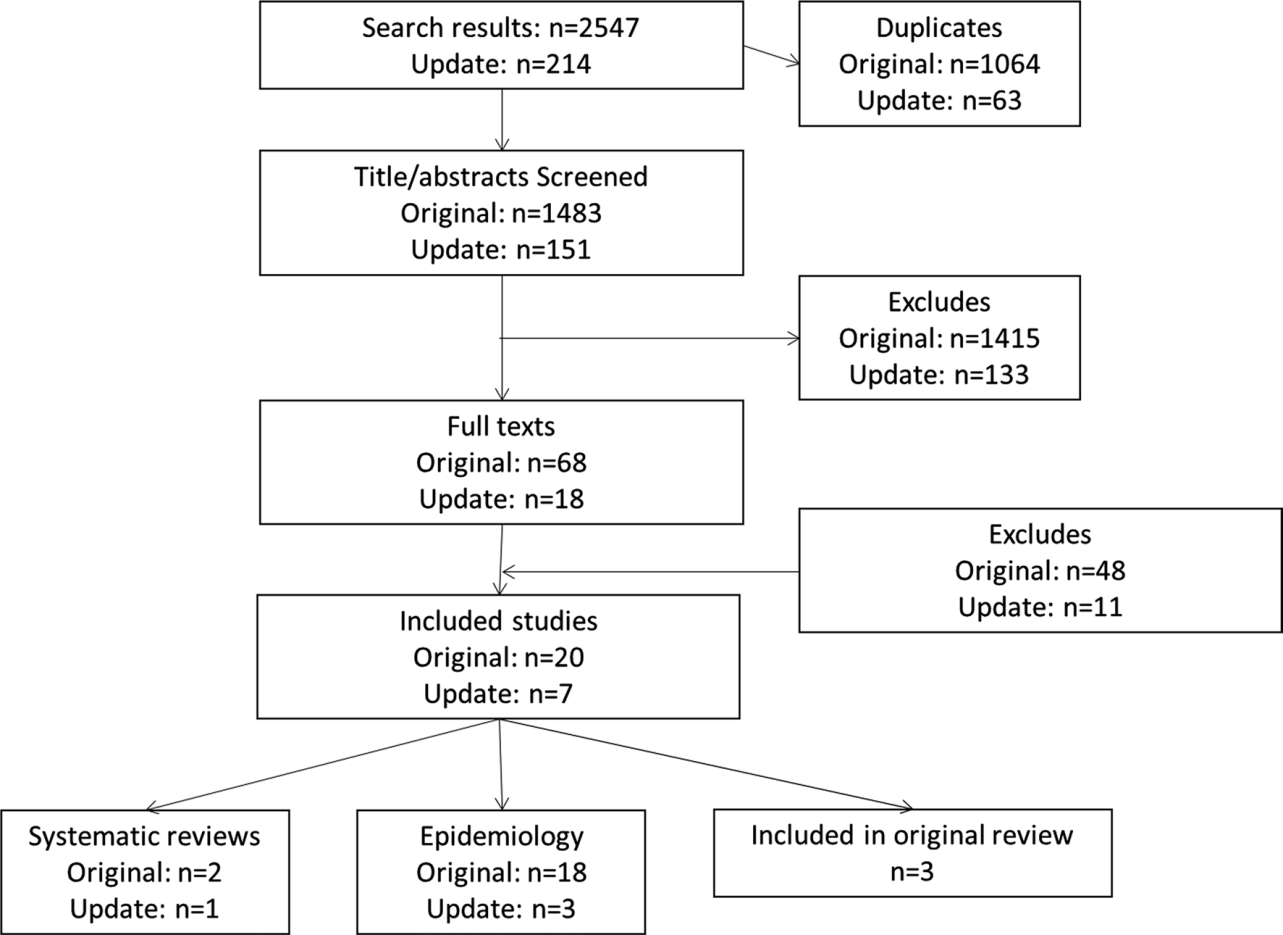
PRISMA flowchart of included studies. *Notes*: The 3 systematic reviews included Baker et al. 2019, which provided the basis for this update review; Davis et al. 2009 and Verkade et al. 2020, where included studies were scrutinised but no additional studies were identified for inclusion [5, 7]

From 1483 abstracts, 68 studies were screened at full text (Fig. 1). Of these, 18 studies were eligible for inclusion. All studies focus on PFIC, with just one reporting PFIC and BRIC. In addition two systematic reviews: Baker et al. 2019 (the basis for this update review, studies published prior to 2015), and Davis et al. 2009 were identified [5, 7]. The latter systematic review included 11 studies focused on nontransplant surgical interventions in PFIC. Since these studies were published before 2015, they were not eligible for screening.

An update search identified seven full texts for inclusion. One of these was a systematic review (Verkade et al. 2020) [8] and three were included in the primary search (Foroutan et al. 2020; Van Vaisberg et al. 2020; Van Wessel et al. 2020), leaving three unique studies [9–11].

The systematic review authored by Verkade et al. 2020 focused on SBD in PFIC with a final yield of 16 publications. Of the post-2015 studies (due to the date limit being 2015 for this review following on from Baker et al. 2019), no includable studies were identified.

### Characteristics of included studies

Of the studies identified as eligible for inclusion (Additional file 1), two were prospective studies with one investigating the safety and efficiency of sertraline in refractory cholestatic pruritus, and the other evaluating outcomes in children with PFIC following LT [12, 13].

The remaining 18 studies were retrospective studies. Of these, six publications reported aspects of the NAPPED study, which aimed to (i) define the natural course of disease in PFIC1 and PFIC2 patients, (ii) define the change in natural course of disease following BD and LT and (iii) establish potential surrogate endpoints [14–19].

Five retrospective studies examined the impact of surgical interventions on disease progression, with one study focusing on PFIC3 [20], one reporting PFIC and BRIC and the others investigating a range of PFIC subtypes [21–24].

The studies reported by Malik et al. 2017 and Meena et al. 2017 reported prevalence in patients seen in their respective institutes in Pakistan and India [25, 26].

Disease progression was examined specifically in Byler disease by Morris et al. 2015 [27] and *ABCB4* genotypes by Schatz et al. 2018 [28].

Finally, Ruth et al. 2018 observed disease progression and mortality for patients with a genetic or phenotypic diagnosis of PFIC and BRIC [29].

Where reported, study periods varied widely, ranging from 3 years to estimate frequency of hepatobiliary disorders [25] to 33 years for genotype/phenotype correlation with clinical course and medical/surgical intervention [29].

Sample size was predictably small given the rarity of PFIC. However, the NAPPED study which included 22 centres across, Europe, North-America, Asia and Australia achieved a sample size of 264 patients, depending on genotype under investigation [19].

The updated search identified three studies [30–32]. Bjornland et al. 2020 report outcomes after SBD over a 25-year period in a population of 26 million, where 24 patients presented with PFIC. Thompson et al. 2020 focus on control of SBA for patients with nontruncated BSEP mutation following treatment with maralixibat (n = 19) [31] whereas Alhebbi et al. 2020 report a 22 year, single centre experience of familial cholestatic genetic disorders, with PFIC 1 to 4 being the most common [30, 31].

Description of patient characteristics was variable due to the methods of identification chosen, but also due to the evolving understanding of the disease (Additional file 2). Where reported, the proportion of males ranged from 38 to 77%. This variability was unsurprising since this is a rare disease that affects both sexes equally [3]. Age of presentation is from newborn to 18 years, which reflected the varying phenotypes of PFIC and BRIC. The type of PFIC under investigation also varied across studies. The NAPPED study was reported in six publications. Four of these focused on PFIC1 (described as FIC1 deficiency) and two on PFIC2 (described as BSEP deficiency) (Van Wessel, 2018 to 2020) [14, 15, 18, 19].

### Quality appraisal of included studies

Table 2 displays the quality appraisal of included studies. Due to the rarity of the disease, studies looking to compare interventions were often underpowered. This was less of an issue for the purposes of understanding disease progression and epidemiology; therefore, the criterion of sample size was generally assessed as not applicable. It should be noted, however, that many publications were abstracts, therefore full details were not available, and the publications could not be appraised.

**Table 2:** Critical appraisal of full texts using Joanna Briggs Institute (JBI) tool for epidemiological studies

	Bjornland, 2020[32]	Flores, 2018[21]	Malik, 2017[25]	Morris, 2015[27]	Schatz, 2018[28]	Thebaut, 2017[12]	Van Vaisberg, 2019[23]	Wang, 2017[24]
Was the sample frame appropriate to address the target population?	Yes	Yes	Yes	Yes	Yes	Yes	Yes	Yes
Were study participants sampled in an appropriate way?	Unclear	Yes	Yes	Yes	Yes	Unclear^b^	Yes	Yes
Was the sample size adequate?	NA	NA	NA	NA	NA	NA	NA	NA
Were the study subjects and the setting described in detail?	Yes	Yes	Yes	Yes	Yes	Yes	Yes	Yes
Were valid methods used for the identification of the condition?	Unclear	Yes	No^a^	Yes	Yes	Unclear^c^	Unclear	Yes
Was the condition measured in a standard, reliable way for all participants?	Unclear	Yes	Yes	Yes	Yes	Yes	Yes	Yes
Was there appropriate statistical analysis?	Yes	Yes	NA	NA	NA	Yes	Yes	NA
Was the response rate adequate, and if not, was the low response rate managed appropriately?	NA	NA	NA	NA	Yes	Yes	NA	NA

^a^Genetic analysis not available

^b^Prospective design, unclear if sampling is consecutive

^c^Unclear if genetic analysis performed on all patients

For retrospective studies where non-response and drop-out were not likely, the criterion of response rate was also assessed as not applicable. Similarly, for this study design, where it was not explicitly mentioned that all patients falling under inclusion criteria were selected, then the assessment was judged as unclear.

For diagnosis, where genetic analysis was not available or it was unclear whether the whole population was tested, the response was also unclear.

Regarding statistics for the outcomes relevant to this review, the results were generally presented as proportions with no statistical analysis performed.

### Evaluation

Additional file 3 displays prevalence and mortality reported in the included studies. It is clear these data are limited. None of the identified studies reported incidence.

#### Prevalence

Prevalence was reported in four studies on a local population level in USA, Pakistan, India and Saudi Arabia (Flores, 2018; Malik, 2017; Meena, 2017; Alhebbi, 2020) [21, 25, 26, 30]. Malik et al. 2017 observed that 1.87% of patients admitted over three years to their Department at The Children’s Hospital & The Institute of Child Health, Lahore were diagnosed with PFIC. Flores et al. 2018 report 17 patients admitted with PFIC from January 1996 to December 2016 and Meena et al. 2017 report PFIC (n = 15) to be the commonest cause of PILBD in infants following 632 biopsies [21, 26]. Alhebbi et al. 2020 note that PFIC1-4 diseases were the major cause of familial liver disorders in their study population with a high consanguinity rate of > 50% [30].

Methods of reporting and diagnosis make comparisons challenging; however, the following studies with prevalence data included in Baker et al., 2019[5] were 11.7% of children with chronic intrahepatic cholestasis across 16 centres in the USA (Kamath, 2015) [33], 12.9% of infants admitted with neonatal cholestasis in Sweden (Fischler, 2001) [34], and 9.0% of infants < 2 years of age with cholestasis, acute liver failure, or splenomegaly (Ruth, 2014) in 13 international centres [35].

#### Mortality

Mortality was generally reported following liver transplant. For example, Varamparampil et al. 2018 reported one-year graft and patient survival was 84% which was significantly lower than children with biliary artresia (BA) and Varamparampil et al. 2019 observed reduced survival in PFIC1 following LT compared to PFIC2 to PFIC4 (63% compared to 84.6%) [13, 22]. In contrast, one study observed that for PFIC3, living-donor LT for PFIC3 has favourable outcome with 100% survival at three years follow-up (Acar, 2019) [20]. Van Vaisberg et al. 2019 reported one death following ileal exclusion.

The NAPPED study, reported in six publications, focused on pre-transplant mortality, which was initially 2% for FIC1-deficiency and 5% for BSEP-deficiency [14, 15, 19]. This was noted to be 9% in a more recent publication for PFIC1 [18].

Ruth et al. 2018 noted earlier presentation of disease was found to be significantly associated with mortality (p < 0.01) for PFIC1 [29].

Comparing PFIC types with BA, one study reported 1-year patient survival rates after transplant to be significantly lower (63%) in PFIC1 compared to PFIC2/3/4 (84.6%) or BA (91%) (Valamparampil, 2019) [22].

Mortality was similarly variable in the Baker et al. 2019 review, with reports between 0 and 87% across 10 studies, reflecting differences in study design, treatment and severity of disease [36–45].

#### Disease progression

Disease progression, which generally leads to surgery such as BD or LT, is described in Additional file 4. For all PFIC types combined, Valamparampil et al. 2018 observed the median age at LT was 46 months (range 6–204 months) and the duration of hospitalisation was 21 days. Incidental hepatocellular carcinoma was noted in explants in four children (16%) which was significantly higher than BA explants [13].

In contrast, for PFIC1, one study reported no evidence of progressive liver disease manifested by features of portal hypertension and/or uncorrectable coagulopathy in their cohort of six patients over seven years (Morris, 2015) [27].

Following SBD, Bjornland et al. 2020 observe PFIC2 patients to have reduced SBA with only 11% progressing to LT, in contrast to PFIC 1, where present SBA levels were greater than pre-operative and 50% of the population received a transplant [32]. Only one patient presented with PFIC3; however, they also progressed to LT. Similarly, Thompson et al. 2020 report patients with BSEP deficiency who achieved control of SBA during MRX treatment had native liver survival beyond 4.5 years, improved liver biochemistry and improved growth [31].

Ruth et al. 2018 reported no significant associations between age of presentation and the need for transplant or medical intervention; however, patients with PFIC1 were more likely to require SBD or LT, 37.5% and 75% respectively [29]. The leading indication for transplant with genetically confirmed PFIC was observed to be progressive liver disease with intractable pruritus. Patients with BRIC typically presented in adolescence, with identified triggers including medications.

Two studies focused on PFIC3 with one reporting indications for liver transplantation to be portal hypertensive bleeding, severe itching, growth failure and cirrhosis in PFIC3 (Acar, 2019) and another observing 16 out of 26 children listed for liver transplantation at a median age of 6.8 years with two deaths due to LT-related complications (Schatz, 2018) [20, 28].

With regard to surgery, one study noted that two patients with PFIC underwent a partial external biliary diversion (PEBD) which was closed because it was not efficient (Thebaut, 2017) [12]. In contrast, Wang et al. 2019 reported a significant reduction in serum total bilirubin following SBD in FIC1 patients but not in BSEP patients [24]. However, symptomatically FIC1 and BSEP patients experienced less pruritus after SBD.

Valamparampil et al. 2018 and Valamparampil et al. 2019 reported outcomes following LT to be inferior in PFIC1 owing to steatosis, post-LT diarrhoea and growth failure as compared to other types of PFIC [13, 22].

Vaisberg et al. 2019 explored the clinical outcomes of patients receiving ileal exclusion (IE) with a follow-up of 60 months [23]. The authors concluded that this procedure provided excellent results in pruritus control and permitted survival with the native liver, with only 25% of patients progressing to ESLD.

Regarding the NAPPED study, 68% of patients with FIC1 deficiency had undergone a surgical biliary diversion (SBD) by 18 years old [18]. Interestingly, an increase of SBD was noted in the second decade of female patients.

At 18 years old, only 38% of patients with FIC1 deficiency were alive with their native liver. SBD was associated with a significant decrease in the levels of serum bile acid (SBA) but was not associated with native liver survival (NLS) (HR = 1.64; 95%CI [0.58–4.60]; p = 0.35), and neither were post-SBD SBA or ALT-levels. Nevertheless, patients with a post-SBD SBA level < 100 mmol/L had significantly higher NLS compared to patients with post-SBD SBA levels of 100 mmol/L (at five years post-SBD, 100% vs. 30%; p = 0.02) [18].

Overall SBD rates at ten years of age were 43%, with SBD rates in compound heterozygous and homozygous patients of 37/45%, respectively (p = 0.29). Overall ten-year NLS was 46%, however, NLS did not differ significantly between patients who had received SBD patients and those who had not [HR = 1.16; 95%CI (0.33–4.01); p = 0.82]. HCC was not encountered in any patient during follow-up, in contrast to observations in other cholestatic diseases.

For the BSEP-deficient population observed in the NAPPED study, there are slight changes in results over time as expected. Therefore, the most recent publication of Van Wessel et al. 2020 is reported in the narrative; however, all results are presented in Additional file 4. For BSEP-deficiency, genotype severity was strongly associated with native liver survival, falling from a median of 20.4 years for BSEP1 to 3.5 years for BSEP3 (p < 0.001) [14, 19]. In terms of disease progression, a similar trend was seen for HCC at 15 years of age, with the proportion of patients increasing from 4% in BSEP1 to 34% in BSEP3 (p = 0.001). SBD was associated with significantly increased NLS (hazard ratio 0.50; 95% CI 0.27–0.94; p = 0.03) in BSEP1 and BSEP2. An SBA concentration below 102 µmol/L or a decrease of at least 75%, shortly after SBD, reliably predicted NLS of ≥ 15 years following SBD (each p < 0.001).

The trend for BSEP-deficiency (or PFIC2) patients to be more likely than patients with PFIC1 or indeed PFIC3 to experience progression to severe liver disease or HCC and to require LT is generally supported by Baker et al. 2019 [5].

### Research question 2: burden of disease

**Fig. 2:**
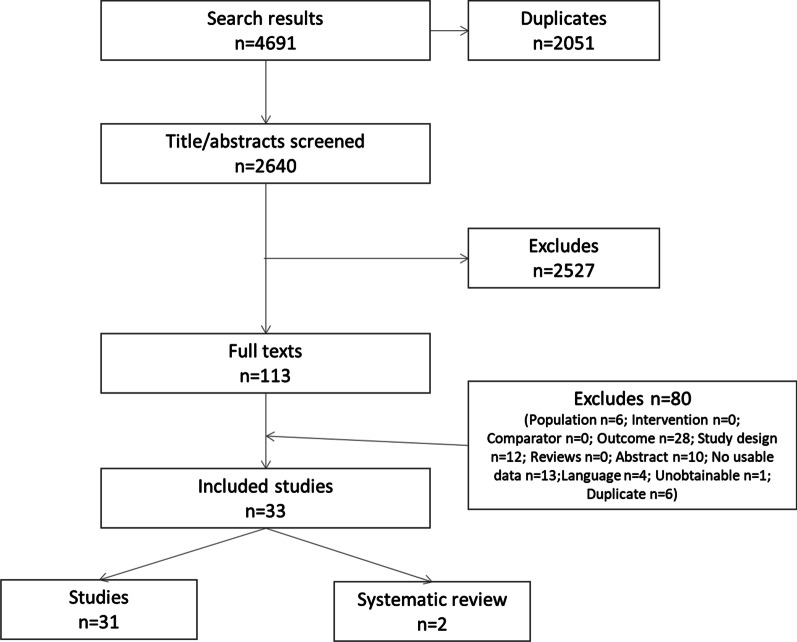
PRISMA flowchart of included studies. *Notes*: The 2 systematic reviews were scrutinised, however, no additional studies identified for inclusion

From 2,640 abstracts identified in the update searches, 113 studies were screened at full text (Fig. 2). Of these, 33 studies were eligible for inclusion.

All studies focus on PFIC, with just one reporting PFIC and BRIC. Ten of the retrieved studies were abstracts and 21 were full texts. In addition to the 31 included studies, two systematic reviews were identified: Davis et al. (2009) and Baker et al. (2019) [5, 7]. None of the outcomes of the studies included in the Davis (2009) review were eligible for inclusion and of the five studies identified as investigating human or economic burden in Baker (2019), none were includable in this review, either because they were only available as abstracts published before 2018, or because the results were not presented in isolation for PFIC.

Following an update search, no new studies were identified on the burden of PFIC.

### Characteristics of included studies

Of the studies identified as eligible for inclusion (Additional file 5), ten were prospective studies with two investigating HRQL [46, 47], seven evaluating surgery rates [9, 49–54]and one reporting patient/graft survival [54]. The remaining 21 studies were retrospective. One of these studies report BRIC and PFIC [29]. Five publications reported aspects of the NAPPED study, which aimed to (i) define the natural course of disease in PFIC1 and PFIC2 patients, (ii) define the change in natural course of disease following BD and LT and (iii) establish potential surrogate endpoints [14, 15, 18, 55].

In terms of outcomes relevant to this review, the remainder of the retrospective studies reported rates of surgery (both LT and BD) and/or survival.

Where reported, study periods varied widely, ranging from one year for a prospective study of UDCA treatment to a 60-year study of population analysis in Greenland.

Sample size was predictably small given the rarity of PFIC. However, the NAPPED study which included 22 centres across Europe, North America, Asia and Australia reported a sample size of 234 patients, depending on genotype under investigation.

Description of patient characteristics was variable due to progress in identifying PFIC subtypes (Additional file 6). Where reported, the proportion of male participants ranged from 32 to 77%. Although this is a disease that affects both sexes equally, the wide range in proportion of male participants is expected due to small sample sizes [3]. Age of presentation was from newborn to 17 years, again reflecting the differing phenotypes of PFIC and BRIC.

PFIC subtypes vary widely, in terms of those selected for inclusion and those where differentiation is recorded. Only one study reports PFIC and BRIC, also noting new genotypes (*TJP2* and *DCDC2*) [29]. The NAPPED study with five abstracts investigates PFIC 1 (described as FIC1 deficiency) and PFIC2 (described as BSEP deficiency) both together and in isolation [14, 15, 18, 55]. Seven studies did not differentiate type of PFIC [46, 48–51, 56, 57] and the remainder examined one or a number of identified types.

### Quality appraisal of included studies

For retrospective studies where non-response and drop-out were not likely, the criterion of response rate was assessed as not applicable (Table 3). Similarly, for this study design, where it was not explicitly mentioned that all patients falling under inclusion criteria were selected, then the assessment was judged as unclear.

**Table 3:** Critical appraisal using Joanna Briggs Institute (JBI) tool for prevalence studies

	Agarwal, 2016[61]	Arnell, 2008[48]	Aydogdu, 2007[49]	Bull, 2018[58]	Chen, 2018[63]	Dinler, 1999[54]	Emond, 1995[50]	Englert, 2007[38]	Erginel, 2018[64]
Was the sample frame appropriate to address the target population?	Yes	Yes	Yes	Yes	Yes	Yes	Yes	Yes	Unclear
Were study participants sampled in an appropriate way?	Yes	Yes	Yes	Yes	Yes	Unclear	Yes	Unclear	Unclear
Was the sample size adequate?	NA	NA	NA	NA	NA	NA	NA	NA	NA
Were the study subjects and the setting described in detail?	Yes	Yes	Yes	Yes	Yes	No	Yes	Yes	Yes
Were valid methods used for the identification of the condition?	Yes	Yes	Yes	Yes	Yes	Yes	Unclear	Unclear	Yes
Was the condition measured in a standard, reliable way for all participants?	Yes	Yes	Yes	Yes	Yes	Yes	Unclear	Unclear	Yes
Was there appropriate statistical analysis?	Yes	Yes	Yes	Yes	Yes	Yes	Yes	NA	Yes
Was the response rate adequate, and if not, was the low response rate managed appropriately?	NA	NA	NA	NA	NA	NA	NA	NA	NA

**Table 4:** Mean PedsQL score for patient with PFIC after LT and PEBD

PedsQL Scale	LT child	LT parent proxy	PEBD child	PEBD parent proxy	p-value
*Mean Score (SD)*					
Total score	77 (16)	84 (13)	80 (14)	81 (17)	ns
Physical health	81 (18)	88 (11)	91 (11)	88 (13)	0.07
Psychosocial health	77 (17)	82 (16)	76 (15)	79 (19)	ns
Emotional functioning	77 (24)	82 (22)	65 (23)	76 (16)	ns
Social functioning	81 (23)	87 (20)	82 (11)	83 (23)	ns
School functioning	72 (17)	79 (14)	82 (16)	83 (15)	ns

*LT* liver transplant, *PEBD* partial external biliary diversion, *PFIC* progressive intrahepatic cholestasis

*Notes*: Physical health, p = 0.07 LT child versus PEBD child

Source: Wassman et al. 2018

For diagnosis, where there the diagnostic criteria were not clear or not available it was rated as unclear.

It should also be noted that for the outcomes relevant to this review, results were generally presented as proportions with no statistical analysis performed (Table 4).

### Evaluation

Additional file 7 displays burden of disease outcomes, largely focusing on HRQL and rates of surgery. Survival has also been included where reported.

Two studies reported HRQL outcomes [46, 47]. Wassman et al. (2018) reported PedsQL generic scores for patients with PFIC after LT and PEBD, where higher scores indicated better HRQL (Additional file 7) [46] A significantly lower mean score in school functioning in the LT group was observed when compared with healthy children, whereas the PEBD group showed no impairment in school functioning, despite having a similar history of cholestatic liver disease. The authors suggested the impact of calcineurin inhibitors may be responsible, since they are known to affect the cognitive functioning of children after LT. This was supported by the observation that PFIC patients living with their native liver did not have poorer HRQL scores than the healthy controls.

The authors also acknowledged that PEBD has the disadvantage of a permanent stoma [46]. When asked about their annoyance by the presence of a permanent stoma, 20% of the patients stated that they never felt annoyed by the stoma. However, another 20% reported always feeling annoyed by the stoma, which suggests a wide variety in stoma acceptance. That said, the small sample size meant that these results should be treated with caution.

Regarding study limitations, the PedsQL has not been validated for patients with PFIC [46]. Several important medical aspects, such as stomata or stigmatising scars, and everyday aspects such as the possibility of pursuing certain hobbies like swimming, were not included in the survey. Another limitation is that the mean age in the LT group was different from that of the PEBD group and the PFIC subtypes were not differentiated.

The second study (Yee, 2018) reporting HRQL observed that LT was associated with more frequent post-surgery complications than BD [47]. Patients who underwent BD all experienced improvements in HRQL, mainly due to improved sleep (73.4%), improved mood (67.4%) and less itching (63.3%). In contrast, a major problem with LT is exacerbation of diarrhoea, which may impair quality of life and may prevent the catch-up growth after transplantation especially in patients with PFIC1 [49].

With regards to rates of surgery for PFIC, most studies focused on BD or LT. Chen et al. (2018), however, reported a 14-year review of cholecystolectomy, which was associated with lower rates of LT and mortality. Of 15 patients, however, four patients (three with PFIC1 and one with PFIC2) experienced recurrence of cholestasis and two underwent reoperation. Two PFIC2 patients underwent LT. One patient with PFIC2 and one patient with PFIC3 died due to severe diarrhoea and dehydration; one PFIC2 patient died of intractable constipation.

Jankowska et al. (2014) observed nine patients who underwent IE [52]. In four children, it was the primary operation (Group 1) and in five children, IE was performed after PEBD (Group 2). However, the authors concluded IE was not as effective as PEBD in long-term outcome and was not recommended as a first-line treatment. In Group 1, one patient was converted to PEBD and in Group 2, one patient went on to LT.

The rates of BD and LT vary widely depending on study period, sample size and PFIC type, with many patients having multiple surgeries and progressing to LT. As such the data are not suitable for comparison. There are, however, several studies of note. Bull et al. (2018) observed 145 patients with FIC1 (PFIC1) or BSEP (PFIC2) undergoing PEBD, IE or LT [58]. Of the FIC1 and BSEP patients, 36% and 42% underwent PEBD, respectively and 42% and 58% underwent LT, respectively. It was also reported that BSEP patients came to LT younger than FIC1 patients.

Where poor outcomes include death, development of cirrhosis, LT, or listing for LT, patients with a PFIC subtype (BSEP-other mutation) fared worse than patients in other post-PEBD groups [58].

Several studies reported that PEBD leads to LT if performed when cirrhosis is established (Emond, 1995; Halaweish, 2010; Englert, 2007; Schukfeh, 2012) [38, 50, 57, 59]. Indeed, Englert et al. (2007) noted that after BD, nearly 60% of the children received LT secondarily because of intractable pruritus or increasing liver cirrhosis. Similarly, Schukfeh et al. 2012 demonstrated excellent long term outcomes of PEBD in PFIC patients without cirrhosis with only 12% needing LT during long-term follow up [57]. Patients with cirrhosis, however, displayed a long-term failure rate of 100%.

While Halaweish et al. 2010 discuss the numerous variations of SBD developed in an attempt to prolong the interval to liver transplantation and, potentially to avert the need for transplantation, they suggest alternative considerations might include IE or PIBD [59]. They warned that whilst IE is initially effective in reducing hyperbilirubinemia-associated pruritus, when it is compared with SBD it does not provide good long-term resolution of symptoms in most patients. This is possibly because of eventual intestinal adaptation leading to increased bile acid reabsorption.

Finally, to summarise the NAPPED study, for PFIC2 (BSEP-def) patients, NLS was significantly higher in patients receiving SBD than in those who did not (HR = 0.44; 95%CI 0.23–0.88, p = 0.007) after adjustment for age and genotype category. Only one-third of PFIC2 patients reach adulthood with their native liver. The effect of surgical biliary diversion, native liver survival and the incidence of HCC were all associated with the severity category of the PFIC2 mutation. Native liver survival in PFIC1 (FIC1-def) patients did not differ significantly between the SBD positive and SBD negative groups [14, 15, 18, 55].

SBD rates at five/ten years of age were 33/39% in PFIC1-def and 28/35% in PFIC2-def patients. Five/ten-year NLS was 73/51% in PFIC1 and 61/46% in PFIC2 patients. Before the age of five/ten years, 27/49% of PFIC1 and 36/52% of PFIC2 patients had been transplanted. Hepatocellular carcinoma was not seen in PFIC1, but in 10% of PFIC2 patients (21% in severe, 12% in medium and 2% in mild mutations; p = 0.006] [14, 15, 18, 55].

SBD rates with age also decreased significantly between patients with mild, medium or severe PFIC2 mutations, as shown by the percentage of patients with SBD at age 10 years: 57%, 21%, and 14%, respectively [14, 15, 18, 55]. Similarly, NLS with age decreased significantly in the order of mild, medium and severe PFIC2 mutations (10 years percentage: 60%, 37%, and 32%, resp.; P < 0.001). The observed NLS at 18 years of age for PFIC1 and PFIC2 was 51% and 32%, respectively.

## Discussion

The studies identified in this review were generally limited by nature of the low incidence of PFIC, paucity of data, variability in study design, and methods of diagnosis. For example, while genetic mutations are continuing to be discovered, there are some cases of PFIC where no mutation at all is identified, despite having GGTP levels appropriate for the condition. Furthermore, the current understanding is that PFIC and BRIC exist on the same spectrum and are not distinct, meaning there may be some crossover in the data. This review, however, adds insight to PFIC and BRIC disease progression, following on from Baker et al. 2019 [5] in particular, where selected treatments and/or surgery may be more suitable to one form of PFIC and at a specific point in the progression of the disease over another. In addition, this review includes a detailed summary of the NAPPED study, which continues to add crucial data to aid our understanding [14, 15, 18, 19, 55, 60].

In general, despite the challenges associated with a rare and complex disease, reporting was clear regarding epidemiology and the studies were well-designed for the outcomes of interest. Due to the retrospective nature of the studies, however, where HRQL data were not routinely collected, there is minimal information for this critical outcome. Finally, many authors acknowledge the studies were underpowered and statistical analysis was limited due to intra-study variability of populations.

### Research question 1: epidemiology

The most detailed data come from the NAPPED study where key findings for FIC1 were that SBD was not significantly associated with NLS, except in for patients in whom SBA decreased to a level below 100 mmol/L after SBD. They found mortality to be relatively low, the majority of patients reaching adulthood with native or transplanted liver [18]. In contrast, Valamparampil et al. 2018 and 2019 found one-year patient survival rates were significantly lower in PFIC1 compared to PFIC2/3/4. Indeed, they amended their unit policy to simultaneous internal biliary diversion along with LT in PFIC1 from 2016 onwards because of inferior graft outcomes [13, 22].

For PFIC2 patients, Bjornland et al. 2020 observed for PFIC2 patients that reduced SBA led to only 11% of patients progressing to LT, in contrast to PFIC 1 where 50% of the population received a transplant [32]. Similarly, Thompson et al. 2020 report patients with BSEP deficiency who achieved control of SBA during MRX treatment had native liver survival beyond 4.5 years, improved liver biochemistry and improved growth [31].

For BSEP-deficiency, Van Wessel et al. 2018 concluded from the NAPPED study that only a third of BSEP patients reached adulthood with their native liver. The effect of SBD, NLS and the incidence of HCC, however, were all associated with the severity category of the BSEP mutation [14, 19]. Native liver survival fell from a median of 20.4 years for BSEP1 to 3.5 years for BSEP3 (p < 0.001) [14, 19]. SBD was associated with significantly increased NLS (hazard ratio 0.50; 95% CI 0.27–0.94; p = 0.03) in BSEP1 and BSEP2. Indeed, an SBA concentration below 102 µmol/L or a decrease of at least 75% shortly after SBD reliably predicted NLS of ≥ 15 years following SBD (each p < 0.001).

Data on PFIC3 appears to be extremely limited, with one study noting that one third of the children with PFIC 3 were initially misdiagnosed, indicating the need for better diagnostic tools and medical education (Schatz, 2018) [28]. Acar et al. 2019, however, found PFIC3 to have a favourable outcome with 100% survival rate over 3 years [20].

There is variability in presentation even within types of PFIC, which will affect the choice of intervention. For example, Morris et al. 2015 discussed the profound cholestasis in the observed cohort with Byler disease and yet a lack of progressive liver disease [27]. As another example of variability within PFIC type, Ruth et al. 2018 noted earlier presentation in PFIC1 was significantly associated mortality (p < 0.01). Yet, there were no significant associations between age of presentation and need for transplant or medical intervention. Patients with PFIC1 were also more likely to require BD or LT, compared to other forms [29].

Wang et al. 2017 noted that surgical interventions which may improve outcomes for one type of PFIC may be less beneficial in another. For example, a significant reduction in serum total bilirubin was observed following PEBD in FIC1 patients but not in BSEP patients. As such, clinicians face many challenges when considering appropriate treatment [24].

### Research question 2: burden

For the two studies which did report HRQL, PEBD appeared to have a minimal impact on quality of life, whereas LT showed a statistically significant impairment in school functioning. The study design, however, had some limitations, in particular a small sample size.

Again, most data derive from the NAPPED study where key findings for FIC1 were that SBD was not significantly associated with NLS, except for patients in whom SBA decreased to a level below 100 mmol/L after SBD. They found mortality to be relatively low with the majority of patients reaching adulthood with native or transplanted liver [55].

For BSEP-deficiency, Van Wessel et al. 2018 concluded that only a third of BSEP patients reached adulthood with their native liver. Furthermore, the effect of SBD, NLS and the incidence of HCC were all associated with the severity category of the BSEP mutation.

## Conclusion

Using robust and transparent methods, this systematic review summarises our current knowledge of PFIC. The epidemiological overview is highly mixed and dependent on presentation and PFIC subtype. Only two studies reported HRQL and mortality results were highly variable across different subtypes. Lack of data and extensive heterogeneity severely limit understanding of this disease area, particularly variation around and within subtypes.

## Supplementary Information

**Additional file 1**. Summary of studies. Study characteristics for research question 1.

**Additional file 2**. Summary of patient characteristics. Patient characteristics for research question 1.

**Additional file 3**. Prevalence and mortality of PFIC in included studies. Results for research question 1.

**Additional file 4**. Studies reporting disease progression. Results for research question 1.

**Additional file 5**. Summary of studies. Study characteristics for research question 2.

**Additional file 6**. Summary of patient characteristics. Patient characteristics for research question 2.

**Additional file 7**. Results for question 2.

## Data Availability

All data generated or analysed during this study are included in this published article [and its supplementary information files].

## Abbreviations

AASLD: American Association for the Study of Liver Diseases
AFP: Alpha-fetoprotein
ALGS: Alagille syndrome
ALP: Alkaline phosphatase
ALT: Alkaline phosphatase
AMCP: Academy of Managed Care Pharmacy
AMSTAR: Assessment of multiple systematic reviews
ATP: Adenosine triphosphate
BA: Biliary atresia
BD: Biliary diversion
BRIC: Benign recurrent intrahepatic cholestasis
BSEP: Bile salt export pump
CADTH: Canadian Agency for Drugs and Technologies in Health
CI: Confidence interval
CINAHL: Cumulative Index to Nursing and Allied Health Literature
CRD: Centre for Reviews and Dissemination
DDW: Digestive Disease Week
EASL: European Association for the Study of Liver Diseases
ECRD: European Conference on Rare Diseases
ESLD: End-stage liver disease
ESPGHAN: European Society for Paediatric Gastroenterology, Hepatology and Nutrition
EURORDIS: European Organisation for Rare Diseases
FIC: Familial intrahepatic cholestasis
FXR: Farnesoid X receptor
GBC: Gallbladder to colon diversion
GGT: Gamma-glutamyl transferase
GGTP: Gamma-glutamyl transpeptidase
HCC: Hepatic cell carcinoma
HR: Hazard ratio
HRQL: Health-related quality of life
HTA: Health technology assessment
HTAi: Health Technology Assessment International
ICD: International Classification of Diseases
ICER: Institute for Clinical and Economic Review
ICP: Intrahepatic cholestasis of pregnancy
IE: Ileal exclusion
ILTS: International Liver Transplantation Society
IQR: Interquartile range
ISOQOL: International Society for Quality of Life Research
ISPOR: International Society for Pharmacoeconomics and Outcomes Research
JBI: Joanna Briggs Institute
LPAC: Low phospholipid-associated cholestasis
LT: Liver transplant
NA: Not applicable
NAPPED: NAtural course and Prognosis of PFIC and Effect of biliary Diversion
NASPGHAN: North American Society for Pediatric Gastroenterology, Hepatology and Nutrition
NICE: National Institute for Health and Care Excellence
NLS: Native liver survival
NORD: National Organisation for Rare Disorders
NR: Not reported
OPTN: Organ Procurement and Transplantation Network
PBAC: Pharmaceutical Benefits Advisory Committee
PEBD: Partial external biliary diversion
PedsQL: Pediatric Quality of Life Inventory
PFIC: Progressive familial intrahepatic cholestasis
PIBD: Partial internal biliary diversion
PICOS: Population, Intervention, Comparator, Outcomes, Study Design
PILBD: Paucity of Interlobular Bile Duct
PRISMA: Preferred Reporting Items for Systematic Reviews and Meta-Analyses
PROSPERO: International prospective register for systematic reviews
PSC: Primary sclerosing cholangitis
RCT: Randomised controlled trial
SBA: Serum bile acid
SBD: Surgical biliary diversion
SD: Standard deviation
SIGN: Scottish Intercollegiate Guidelines Network
SLR: Systematic literature review
UDCA: Ursodeoxycholic acid
UK: United Kingdom
UNOS: United Network for Organ Sharing
USA: United States of America

## References

[1] Gunaydin M, Cil ATB (2018). Progressive familial intrahepatic cholestasis: diagnosis, management, and treatment. Hepat Med.

[2] Pawlikowska L, Strautnieks S, Jankowska I, Czubkowski P, Emerick K, Antoniou A (2010). Differences in presentation and progression between severe FIC1 and BSEP deficiencies. J Hepatol.

[3] Srivastava A (2014). Progressive familial intrahepatic cholestasis. J Clin Exp Hepatol.

[4] Mehl A, Bohorquez H, Serrano M-S, Galliano G, Reichman TW (2016). Liver transplantation and the management of progressive familial intrahepatic cholestasis in children. World J Transplant.

[5] Baker A, Kerkar N, Todorova L, Kamath BM, Houwen RH (2019). Systematic review of progressive familial intrahepatic cholestasis. Clin Res Hepatol Gastroenterol.

[6] The Joanna Briggs Institute. Checklist for prevalence studies [Available from: http://joannabriggs.org/research/critical-appraisal-tools.html.

[7] Davis AR, Rosenthal P, Newman TB (2009). Nontransplant surgical interventions in progressive familial intrahepatic cholestasis. J Pediatr Surg.

[8] Verkade HJ, Thompson RJ, Arnell H, Fischler B, Gillberg PG, Mattsson JP (2020). Systematic review and meta-analysis: partial external biliary diversion in progressive familial intrahepatic cholestasis. J Pediatr Gastroenterol Nutr.

[9] Foroutan HR, Bahador A, Ghanim SM, Dehghani SM, Anbardar MH, Fattahi MR (2020). Effects of partial internal biliary diversion on long-term outcomes in patients with progressive familial intrahepatic cholestasis: experience in 44 patients. Pediatr Surg Int.

[10] Van Vaisberg V, Tannuri ACA, Lima FR, Tannuri U (2020). Ileal exclusion for pruritus treatment in children with progressive familial intrahepatic cholestasis and other cholestatic diseases. J Pediatr Surg.

[11] van Wessel DBE, Thompson RJ, Gonzales E, Jankowska I, Sokal E, Grammatikopoulos T (2020). Genotype correlates with the natural history of severe bile salt export pump deficiency. J Hepatol.

[12] Thebaut A, Habes D, Gottrand F, Rivet C, Cohen J, Debray D (2017). Sertraline as an additional treatment for cholestatic pruritus in children. J Pediatr Gastroenterol Nutr.

[13] Valamparampil J, Shanmugam N, Reddy MS, Rela M. Liver transplantation in progressive familial intrahepatic cholestasis: outcome analysis from a single centre. Transplantation. 2018;102(5 Supplement 1):141–2.

[14] Van Wessel D, Thompson RJ, Grammatikopoulos T, Kadaristiana A, Jankowska I, Lipinski P (2018). The natural course of BSEP deficiency: Results from the global napped-consortium. Hepatol.

[15] Van Wessel D, Thompson RJ, Grammatikopoulos T, Kadaristiana A, Jankowska I, Lipinski P (2018). The natural course of fic1 deficiency: Results from the napped-consortium. Hepatol.

[16] Van Wessel D, Thompson R, Grammatikopoulos T, Kadaristiana A, Jankowska I, Lipinski P (2018). The natural course of FIC1 deficiency and BSEP deficiency: Initial results from the NAPPEDconsortium (NAtural course andprognosis of PFIC and effect of biliary diversion). J Pediatr Gastroenterol Nutr.

[17] Van Wessel D, Thompson R, Grammatikopoulos T, Kadaristiana A, Jankowska I, Lipinski P (2018). The natural course of FIC1 deficiency and BSEP deficiency: Initial results from the NAPPED-consortium (Natural course and Prognosis of PFIC and Effect of biliary Diversion). J Hepatol.

[18] Van Wessel D, Thompson R, Grammatikopoulos T, Kadaristiana A, Jankowska I, Lipinski P (2019). Factors associated with the natural course of disease in patients with FIC1-deficiency: The NAPPED-consortium. J Pediatr Gastroenterol Nutr.

[19] van Wessel DBE, Thompson RJ, Gonzales E, Jankowska I, Sokal E, Grammatikopoulos T, et al. Genotype correlates with the natural history of severe bile salt export pump deficiency. Journal of Hepatology. 2020. 10.1016/j.jhep.2020.02.007 32087350

[20] Acar S, Demir B, Ayyildiz H, Polat KY, Kanmaz T, Akyildiz M (2019). Living donor liver transplantation for PFIC type 3. J Pediatr Gastroenterol Nutr.

[21] Flores CD, Yu YR, Miloh TA, Goss J, Brandt ML (2018). Surgical outcomes in alagille syndrome and PFIC: a single institution's 20-year experience. J Pediatr Surg.

[22] Valamparampil JJ, Rinaldhy K, Reddy MS, Shanmugam N, Rela M (2019). Outcomes of liver transplantation for pediatric recipients with progressive familial intrahepatic cholestasis. J Clin Exp Hepatol.

[23] Van Vaisberg V, Tannuri ACA, Lima FR, Tannuri U. Ileal exclusion for pruritus treatment in children with progressive familial intrahepatic cholestasis and other cholestatic diseases. J Pediatr Surg. 2019. 10.1016/j.jpedsurg.2019.09.018 31708211

[24] Wang KS, Tiao G, Bass LM, Hertel PM, Mogul D, Kerkar N, et al. Analysis of surgical interruption of the enterohepatic circulation as a treatment for pediatric cholestasis. Hepatol. 2017;(no pagination). 10.1002/hep.29019 PMC5397365 28027587

[25] Malik HS, Cheema HA, Hashmi MA, Waheed N, Anwar A, Anjum MN (2017). Frequency of hepato-biliary disorders in children presenting at the children's hospital & the institute of child health, lahore, a tertiary care referral center. Pak Paed J.

[26] Meena BL, Khanna R, Sharma CB, Rawat D, Alam S. Ductal paucity in childhood: spectrum, profile and outcome. Hepatol Int. 2017;11(1 Supplement 1):S366.

[27] Morris AL, Bukauskas K, Sada RE, Shneider BL (2015). Byler disease: early natural history. J Pediatr Gastroenterol Nutr.

[28] Schatz SB, Jungst C, Keitel-Anselmo V, Kubitz R, Becker C, Gerner P (2018). Phenotypic spectrum and diagnostic pitfalls of ABCB4 deficiency depending on age of onset. Hepatol Commun.

[29] Ruth N, Sharif K, McGovern-Weijers A, Hartley J, Van Mourik I, Kelly D (2018). Long term outcome of children with PFIC-A single centre experience. J Pediatr Gastroenterol Nutr.

[30] Alhebbi H, Alhafaf F, Alqahtani A, Algubaisi S, Wali S (2020). 391 Genetically confirmed familial cholestatic liver disorders in Saudi Arabia, a 22 years single center experience. J Pediatr Gastroenterol Nutr.

[31] Thompson RJ, Kelly D, Miethke A, Rajwal S, Soufi N, Jankowska I (2020). LBO08 Serum bile acid control in long-term maralixibat-treated patients is associated with native liver survival in children with progressive familial intrahepatic cholestasis due to bile salt export pump deficiency (S120). J Hepatol.

[32] Bjornland K, Hukkinen M, Gatzinsky V, Arnell H, Pakarinen MP, Almaas R, et al. Partial Biliary Diversion May Promote Long-Term Relief of Pruritus and Native Liver Survival in Children with Cholestatic Liver Diseases. Eur J Pediatr Surg. 2020. 10.1055/s-0040-1714657 32707578

[33] Kamath BM, Chen Z, Romero R, Fredericks EM, Alonso EM, Arnon R, et al. Quality of life and its determinants in a multicenter cohort of children with Alagille syndrome. J Pediat. 2015;167(2):390–6. e3. 10.1016/j.jpeds.2015.04.077 PMC4516587 26059338

[34] Fischler B, Papadogiannakis N, Nemeth A (2001). Aetiological factors in neonatal cholestasis. Acta Paediatr.

[35] Ruth N, Gray Z, McKay K, Lloyd C, Hartley J, MacDonald F (2014). Identifying incidence of inherited metabolic disorders in patients with infantile liver disease. J Hepatol.

[36] Al Mehaidib A, Al SA (2013). 1381 progressive familial intrahepatic cholestasis in ARABS. J Hepatol.

[37] Davit-Spraul A, Fabre M, Branchereau S, Baussan C, Gonzales E, Stieger B (2010). ATP8B1 and ABCB11 analysis in 62 children with normal gamma-glutamyl transferase progressive familial intrahepatic cholestasis (PFIC): phenotypic differences between PFIC1 and PFIC2 and natural history. Hepatol.

[38] Englert C, Grabhorn E, Richter A, Rogiers X, Burdelski M, Ganschow R (2007). Liver transplantation in children with progressive familial intrahepatic cholestasis. Transplantation.

[39] Henriksen N, Drabløs P, Aagenaes O. Cholestatic jaundice in infancy. The importance of familial and genetic factors in aetiology and prognosis. Arch Dis Childh. 1981;56(8):622–7. 10.1136/adc.56.8.622 PMC1627279 7271301

[40] Miyagawa-Hayashino A, Egawa H, Yorifuji T, Hasegawa M, Haga H, Tsuruyama T (2009). Allograft steatohepatitis in progressive familial intrahepatic cholestasis type 1 after living donor liver transplantation. World J Transplant.

[41] Hori T, Egawa H, Takada Y, Ueda M, Oike F, Ogura Y (2011). Progressive familial intrahepatic cholestasis: a single-center experience of living-donor liver transplantation during two decades in Japan. Clin Transplant.

[42] Lee W, Chai P, Looi L (2009). Progressive familial intrahepatic cholestasis in Malaysian patients—a report of five cases. Med J Malaysia.

[43] Nielsen I, Eiberg H (2004). Cholestasis Familiaris Groelandica: an epidemiological, clinical and genetic study. Int J Circumpolar Health.

[44] Wanty C, Joomye R, Van NH, Paul K, Otte J-B, Reding R (2004). Fifteen years single center experience in the management of progressive familial intrahepatic cholestasis of infancy. Acta Gastroenterol.

[45] Whitington PF, Freese DK, Alonso EM, Schwarzenberg SJ, Sharp HL (1994). Clinical and biochemical findings in progressive familial intrahepatic cholestasis. J Pediatr Gastroenterol Nutr.

[46] Wassman S, Pfister ED, Kuebler JF, Ure BM, Goldschmidt I, Dingemann J (2018). Quality of life in patients with progressive familial intrahepatic cholestasis: No difference between post-liver transplantation and post-partial external biliary diversion. J Pediatr Gastroenterol Nutr.

[47] Yee K, Moshkovich O, Llewellyn S, Benjamin K, Desai NK (2018). A web-based survey of itch severity after surgical treatment of progressive familial intrahepatic cholestasis in children and adolescents. Hepatol.

[48] Arnell H, Bergdahl S, Papadogiannakis N, Nemeth A, Fischler B (2008). Preoperative observations and short-term outcome after partial external biliary diversion in 13 patients with progressive familial intrahepatic cholestasis. J Pediatr Surg.

[49] Aydogdu S, Cakir M, Arikan C, Tumgor G, Yuksekkaya HA, Yilmaz F (2007). Liver transplantation for progressive familial intrahepatic cholestasis: clinical and histopathological findings, outcome and impact on growth. Pediatr Transplant.

[50] Emond JC, Whitington PF (1995). Selective surgical management of progressive familial intrahepatic cholestasis (Byler's disease). J Pediatr Surg.

[51] Jacquemin E, Hermans D, Myara A, Habes D, Debray D, Hadchouel M (1997). Ursodeoxycholic acid therapy in pediatric patients with progressive familial intrahepatic cholestasis. Hepatol.

[52] Jankowska I, Czubkowski P, Kalicinski P, Ismail H, Kowalski A, Ryzko J (2014). Ileal exclusion in children with progressive familial intrahepatic cholestasis. J Pediatr Gastroenterol Nutr.

[53] Wanty C, Joomye R, Van Hoorebeek N, Paul K, Otte JB, Reding R (2004). Fifteen years single center experience in the management of progressive familial intrahepatic cholestasis of infancy. Acta Gastroenterol.

[54] Dinler G, Kocak N, Ozen H, Yuce A, Gurakan F (1999). Ursodeoxycholic acid treatment in children with Byler disease. Pediatr Int.

[55] van Wessel D, Thompson R, Grammatikopoulos T, Kadaristiana A, Jankowska I, Lipin?ski P, et al. Predicting long-term outcome after surgical biliary diversion in Bsep-deficiency patients: Results from the NAPPED consortium. J Hepatol. 2019;70(1):e121.

[56] Ismail H, Kalicinski P, Markiewicz M, Jankowska I, Pawlowska J, Kluge P (1999). Treatment of progressive familial intrahepatic cholestasis: liver transplantation or partial external biliary diversion. Pediatr Transplant.

[57] Schukfeh N, Metzelder ML, Petersen C, Reismann M, Pfister ED, Ure BM (2012). Normalization of serum bile acids after partial external biliary diversion indicates an excellent long-term outcome in children with progressive familial intrahepatic cholestasis. J Pediatr Surg.

[58] Bull LN, Pawlikowska L, Strautnieks S, Jankowska I, Czubkowski P, Dodge JL (2018). Outcomes of surgical management of familial intrahepatic cholestasis 1 and bile salt export protein deficiencies. Hepatol Commun.

[59] Halaweish I, Chwals WJ (2010). Long-term outcome after partial external biliary diversion for progressive familial intrahepatic cholestasis. J Pediatr Surg.

[60] Van Wessel D, Thompson R, Grammatikopoulos T, Kadaristiana A, Jankowska I, Lipinski P (2019). Predicting long-term outcome after surgical biliary diversion in BSEP-deficiency patients: Results from the NAPPED consortium. J Pediatr Gastroenterol Nutr.

[61] Agarwal S, Lal BB, Rawat D, Rastogi A, Bharathy KGS, Alam S (2016). Progressive familial intrahepatic cholestasis (PFIC) in Indian Children: clinical spectrum and outcome. J Clin Exp Hepatol.

[62] Cantez MS, Onal Z, Guller D, Ekici F, Gulluoglu M, Soysal FG (2018). Diverse mutations and different clinical outcomes in children with progressive intrahepatic cholestasis. J Pediatr Gastroenterol Nutr.

[63] Chen L, Xiao H, Ren X-H, Li L (2018). Long-term outcomes after cholecystocolostomy for progressive familial intrahepatic cholestasis. Hepatol Res.

[64] Erginel B, Soysal FG, Durmaz O, Celik A, Salman T (2018). Long-term outcomes of six patients after partial internal biliary diversion for progressive familial intrahepatic cholestasis. J Pediatr Surg.

[65] Nielsen IM, Eiberg H (2004). Cholestasis Familiaris Groenlandica: an epidemiological, clinical and genetic study. Int J Circumpolar Health.

[66] Varma S, Revencu N, Stephenne X, Scheers I, Smets F, Beleza-Meireles A (2015). Retargeting of bile salt export pump and favorable outcome in children with progressive familial intrahepatic cholestasis type 2. Hepatol.

