# Supplementary material for: Epidemiology and burden of progressive familial intrahepatic cholestasis: a systematic review
Source: Orphanet J Rare Dis. 2021 Jun 3;16:255. doi: 10.1186/s13023-021-01884-4 (PMC8173883; doi:10.1186/s13023-021-01884-4)
Supplement: Supplementary file 1 — Additional file 1. Summary of studies. Study characteristics for research question 1. [file 13023_2021_1884_MOESM1_ESM.docx]

**Additional file 1**

**Summary of studies**

| **First author, year** | **Sample size**  **Study design** | **Objective** | **Setting, location** | **Study period** | **Population** | **Relevant outcomes reported** |
| --- | --- | --- | --- | --- | --- | --- |
| **Acar, 2019[21]** | N=13  Retrospective data analysis | To summarize the experience of transplanted children with PFIC3 | Medical records and clinical charts, Turkey | Median follow up, years (range): 3 (0.9-6) | Patients with PFIC type 3 | Disease progression; mortality |
| **Alhebbi, 2020[31]** | N=455 with liver disorders; PFIC 1 to 4: 42.4%  Retrospective | To identify the aetiological causes of familial cholestatic liver disorders proved by genetic analysis. | Hepatology unit, Saudi Arabia | 1998 to 2020 | PFIC 1 to 4: 42.4% | Prevalence |
| **Bjornland, 2020[33]** | N=33  Retrospective | To report long term outcome after SBD during a 25-year period | Hospitals, Norway, Finland, Sweden | 1992 to 2018 | PFIC1: n=4  PFIC2: n=19  PFIC3: n=1 | Disease progression |
| **Flores, 2018[22]** | N=37  Retrospective data analysis | To review the experience with the surgical management of PFIC and ALGS over the last 20 years. | NR, USA | Jan 1996 to Dec 2016 | Patients identified via ICD-9 codes. Patients excluded if not confirmed by imaging, genetics or labs. | Disease progression |
| **Malik, 2017[26]** | N=644  Retrospective descriptive study | To determine the frequency of hepato-biliary disorders among the patients over  three years. | Hospital, Lahore, Pakistan | Jan 2013 to Dec 2015 | Patients with hepato-biliary disorders | Prevalence |
| **Meena, 2017[27]** | N=632 biopsies  Retrospective data analysis | To provide data defining the etiological profile and natural course of non-syndromic PILBD | Institute of Liver and Biliary Sciences, New Delhi, India | NR | All liver biopsies performed in children < 18 years old were screened for ductal paucity | Prevalence; disease progression |
| **Morris, 2015[28]** | N=6  Retrospective analysis | To inform clinical understanding of the early course of hepatic manifestations of Byler disease. | Hospital, USA | Jan 2007 to Oct 2014 | Children with Byler disease (defined by homozygous c.923G>T mutation in *ATP8b1*) | Disease progression |
| **Ruth, 2018[30]** | N=80  Retrospective descriptive study | Genotype/phenotype correlation with clinical course and medical/surgical intervention. | Hospital, Birmingham, United Kingdom, UK | 1984 to 2017 | Patients with a genetic or phenotypic diagnosis of PFIC. | Disease progression; mortality |
| **Schatz, 2018[29]** | N=38  Retrospective collection of clinical and laboratory data | To collect information on onset/progression of ABCB4 gene phenotypes in different age groups and assess relevance for the differential diagnosis of chronic liver disease. | Ten hospitals, Germany | NR | Patients with PFIC, ICP or LPAC syndrome. | Disease progression; mortality |
| **Thebaut, 2017[13]** | N=20 (13 ALGS; 7 PFIC) Prospective | To assess efficiency and safety of sertraline in treating children with refractory cholestatic pruritus | Four paediatric hepatology centres, Germany | June 1, 2007 to May 31, 2014 | Children presenting with chronic refractory cholestatic pruritus | Mortality |
| **Thompson, 2020{Thompson)** | N=19  Prospective open label study  NCT02057718 | To describe >4.5 years of treatment with maralixibat. | NR | >4.5 years | Nontruncated BSEP mutation | Disease progression |
| **Valamparampil, 2018[14]** | N=25 patients with PFIC vs 50 controls Prospective | NR | NR | The median follow-up, years (range): 3.5 (0.5 month - 6.5 years). | Children with PFIC and LT | Disease progression; mortality |
| **Valamparampil, 2019[23]** | N=34  Retrospective data analysis | To determine LT outcomes for recipients with PFIC or BA. | Transplant unit, India | 2010 to 2018 | Patients with PFIC who had received a liver transplant | Disease progression; mortality |
| **Van Wessel, 2018[15]** | N=203  Retrospective follow up (NAPPED) | To better understand the natural course of FIC1-def and the efficacy of interventions. | 22 centres, Europe, North America, Asia and Australia. | NR | Patients with compound heterozygous or homozygous *ABCB11* mutations | Disease progression |
| **Van Wessel, 2018[16]** | N=46  Retrospective follow up (NAPPED) | To understand the natural course of FIC1-def and the efficacy of interventions. | 22 centres, Europe, North America, Asia and Australia. | NR | FIC1 - All patients either compound heterozygous or homozygous for disease associated mutations in the *ATP8B1* gene | Disease progression, mortality |
| **Van Wessel, 2018[15]** | N=226 (42 FIC1-def; 184 BSEP-def)  Retrospective follow up (NAPPED) | To understand the nature of PFIC and the efficacy of interventions | 22 centres, Europe and Asia | NR | Patients who were homozygous or compound heterozygous for disease associated mutations in *ATP8B1* or *ABCB11* | Disease progression; mortality |
| **Van Wessel, 2018[16]** | N= 234 (42 FIC1-def ; 192 BSEP-def)  Retrospective follow up (NAPPED) | To understand the nature of PFIC and the efficacy of interventions | 22 centres, Europe and Asia | NR | Patients who were homozygous or compound heterozygous for disease associated mutations in *ATP8B1* or *ABCB11* | Disease progression; mortality |
| **Van Wessel, 2019[19]** | N=55  Retrospective follow up (NAPPED) | To provide genotypical, phenotypical and biochemical factors associated with SBD and NLS in FIC-1 deficiency | 22 centres, Europe and Asia | 3.2 (1.2-6.1) years | Patients with FIC1 deficiency | Disease progression; mortality |
| **Van Wessel, 2020[20]** | N=264  Multicentre, retrospective cohort study (NAPPED) | To determine outcomes following SBD in the largest genetically defined cohort of patients with severe BSEP deficiency to date. | NAPPED consortium currently comprises 48 tertiary referral centres from all over the globe | 1977 to 2019 | Patients with *ABCB11* categorized according to genotypic severity (BSEP1, BSEP2, BSEP3). | Disease progression, mortality |
| **Van Vaisberg, 2019[24]** | N=11 (8 with PFIC)  Retrospective chart review | To conduct a retrospective chart review of patients submitted to ileal exclusion | Paediatric Liver Transplantation Unit,Sao Paulo, Brazil | 1995 to 2018 | Patients diagnosed with PFIC and non-infectious chronic intrahepatic cholestasis | Disease progression; mortality |
| **Wang, 2017[25]** | N=58 (38 with PFIC)  Retrospective data analysis | To evaluate the efficacy of non-transplant surgery for paediatric cholestasis | The Childhood Liver Disease Research Network and research consortium of 15 North American paediatric centres | 2005 to 2013 | Children with ALGS, FIC1, BSEP disease or indeterminate low GGTP intrahepatic cholestasis (GGTP < 100), who underwent non-transplant surgical intervention for cholestasis. | Disease progression; mortality |

**Abbreviations**: ALGS, Alagille syndrome; BSEP, bile salt export pump; FIC1, familial intrahepatic cholestasis 1; GGTP, gamma-glutamyl transpeptidase; ICD, International Classification of Diseases; ICP, intrahepatic cholestasis of pregnancy; LPAC, low phospholipid-associated cholestasis; LT, liver transplant; NLS, native liver survival; NR, not reported; PFIC, progressive intrahepatic cholestasis; PILBD, Paucity of Interlobular Bile Duct.
