# Supplementary material for: Epidemiology and burden of progressive familial intrahepatic cholestasis: a systematic review
Source: Orphanet J Rare Dis. 2021 Jun 3;16:255. doi: 10.1186/s13023-021-01884-4 (PMC8173883; doi:10.1186/s13023-021-01884-4)
Supplement: Supplementary file 2 — Additional file 2. Summary of patient characteristics. Patient characteristics for research question 1. [file 13023_2021_1884_MOESM2_ESM.docx]

**Additional file 2**

**Summary of patient characteristics**

| **First author, year** | **Sample size**  **Reported population** | **Characteristics** | **Relevant treatment** |
| --- | --- | --- | --- |
| **Acar, 2019[21]** | N=13  Patients with PFIC3 | Median age, years (range) 2.4 (0.8 to 6)  Male (%): 77  PFIC3 (%): 100 | Living donor liver transplant |
| **Alhebbi, 2020[31]** | N=193  Patients diagnosed with familial liver disorders | Age: NR  Male (%): NR  PFIC1/2/3/4 (%): 14/37.3/38.3/10.7 | NA |
| **Bjornland, 2020[33]** | N=33 (24 with PFIC)  NR – patients recruited received SBD for intractable pruritus | Mean age at SBD, years (range) :PFIC1: 0.7 (0.5 to 0.9); PFIC2: 1.6 (0.8 to 1.3); PFIC3: 5.8  Male (%):42  PFIC1/2/3 (%): 17/79/4 | SBD |
| **Flores, 2018[22]** | N=37  Patients were identified using ICD-9 diagnosis codes.  Patients were excluded if not confirmed by imaging, genetics or labs to have ALGS or PFIC or they had not undergone surgical intervention. | Median age at intervention, years: PFIC:2.4 (1.1)  Male (%): 62  PFIC1/2/3 (%): 11/24/11 | 14 patients received a liver transplant; 2 internal diversion (PFIC); 1 external diversion (Alagille). |
| **Malik, 2017[26]** | N=644  Patients with hepato-biliary disorders | Mean age, years (SD): 5.73 (4.31)  Male (%):63  PFIC: Not differentiated | NR |
| **Meena, 2017[27]** | N=632 biopsies  All the liver biopsies performed in children <18 years old were screened retrospectively for evidence of ductal paucity. | Age: NR  Male (%): 77  PFIC2/3 (%): 14/1 | NR |
| **Morris, 2015[28]** | N=6  Children with Byler disease (defined by homozygous c.923G>T mutation inATP8b1) | Age range, days: newborn to 135  Male (%): NR  Byler disease | NR |
| **Ruth, 2018[30]** | N=80  Patients with a genetic or phenotypic diagnosis of PFIC. | Median age, days (range): PFIC1, 213 (12-416); PFIC2, 66 (9-1003); PFIC3, 29 (29-47)  Male (%): 46  PFIC1/2/3 (%): 8/20/2  Unknown (%): 37  BRIC (%): 13 | NR |
| **Schatz, 2018[29]** | N=38  Patients with PFIC, ICP or LPAC syndrome. | Median age of onset, years (range): 0.4 (0.1 to 13.)  Male (%): 45  PFIC3 (%): 100 | 24 patients received UDCA treatment. 9 patients received rifampicin and phenobarbital. |
| **Thebaut, 2017[13]** | N=20 (13 ALGS; 7 PFIC)  Children presenting with chronic refractory cholestatic pruritus | Age range, years: 2.3 to 8  Male (%): 42 (PFIC population)  PFIC2/?: 43/56 | Oral treatment with sertraline UDCA and rifampicin. |
| **Thompson, 2020[32]** | N=19  Patients with nontruncated BSEP deficiency | Median, years (range): 4.1 (1 to 13)  Male (%): 32  Nontruncated BSEP deficiency | Maralixibat |
| **Valamparampil, 2018[14]** | N=25 patients with PFIC; 50 controls  Children with PFIC and LT | Median age of onset, months: 3 (1-47)  Male (%): NR  PFIC 1/2/3/4 (%): 28/28/40/4 | 5 children had undergone previous internal biliary diversion. |
| **Valamparampil, 2019[23]** | N=34  Patients with PFIC who had received a liver transplant | Median age, months:68  Male (%): NR  PFIC1/2/3/4 (%): 23/21/50/6 | Patients received a liver transplant - 94% of total group were from a living donor |
| **Van Wessel, 2018[15]** | N=203  Patients with compound heterozygous or homozygous *ABCB11* mutations | Median age at first visit (range): 9 months (0-195)  Male (%): 53  BSEP-def patients with compound heterozygous or homozygous ABCB11 mutations: mild (n=68), moderate (n=100) or severe (n=35). | UDCA: 47% at first visit |
| **Van Wessel, 2018[16]** | N=46  FIC1 - patients either compound heterozygous or homozygous for disease associated mutations in *ATP8B1* gene | Median age at first visit, years (range): 0.5 (0-16.8)  Male (%):76  FIC1-def: 100 | Use of UDCA prior to first visit 39% |
| **Van Wessel, 2018[15]** | N=226  Patients either homozygous or compound heterozygous for disease associated mutations in *ATP8B1* or *ABCB11* | Median age at first visit, months (range): FIC1-def, 6 (0-201); BSEP-def, 9 (0-195)  Male (%): NR  FIC1-def (%): 19 BSEP-def (%): 81 | NR |
| **Van Wessel, 2018[16]** | N=234 (42 FIC1-def and 192 BSEP-def)  Patientseither homozygous or compound heterozygous for disease associated mutations in *ATP8B1* or *ABCB11* | Median age at first visit, months (range): FIC1-def, 6 (0-201); BSEP-def, 9 (0-195)  Male (%): NR  FIC1 def (%): 18 BSEP def (%): 82 | NR |
| **Van Wessel, 2019[19]** | N=55  Patients with FIC1 deficiency | Median age at first visit, years (range): 0.5 (3-1.1)  Male (%): 77  PFIC1 (%): 100 | NR |
| **Van Wessel, 2020[20]** | N=264  Patients with *ABCB11* categorized according to genotypic severity (BSEP1, BSEP2, BSEP3). | Median age at presentation, years (range): 0.7 (0.2–1.9) Male (%): 50  Patients with *ABCB11* categorised according to genotypic severity (BSEP1, BSEP2, BSEP3) | NR |
| **Van Vaisberg, 2019[24]** | N=11 (8 with PFIC)  Patients diagnosed with PFIC and non-infectious chronic intrahepatic cholestasis | Mean, years (range): PFIC, 5.5 (2 to 14)  Male (%): 50  PFIC1/2/3 (%):62/12/25 | IE during study |
| **Wang, 2017[25]** | N=58 (38 with PFIC)  Children with ALGS, FIC1, BSEP disease or GGTP < 100, with non-transplant surgical intervention for cholestasis. | Age: NR  Male (%):38  FIC1 (%): 42 BSEP (%): 47 GGTP<100 (%): 10 | PEBD: 68%.  IE 15%: 13%  IE 40%: 3%  GBC: 16% |

**Abbreviations:** ALGS, Alagille syndrome; BSEP, bile salt export pump; FIC1, familial intrahepatic cholestasis 1; GBC, gallbladder to colon diversion; GGTP, gamma-glutamyl transpeptidase; ICP, Intrahepatic cholestasis of pregnancy; IE, ileal exclusion; LPAC, low phospholipid-associated cholestasis; LT, Liver transplant; ICP, intrahepatic cholestasis of pregnancy; LT, liver transplant; NR, not reported; PEBD, partial external biliary diversion; PFIC, progressive intrahepatic cholestasis; PFIC?, variant unknown; SBD, Surgical biliary diversion; UCDA, Ursodeoxycholic acid.

| **Van Wessel, 2018[16]** | N = 234  FIC1 def (%): 18  BSEP def (%): 82  Treatment NR | NR | NR | Pre-transplant mortality was 2% for FIC1-def and 4% for BSEP-def. |
| --- | --- | --- | --- | --- |
| **Van Wessel, 2019[19]** | N = 55  PFIC1(%): 100  Treatment: NR | 3.2 (1.2-6.1) years | NR | Prior to LT 9% |
| **Van Wessel, 2020[20]** | N = 264  Patients with ABCB11 according to severity (BSEP1, BSEP2, BSEP3)  Treatment NR | Data collected by investigators within each centre since 1977. Data exported from REDCap on March 1, 2019 | NR | 16 patients (BSEP1 n = 3/72 [4%], BSEP2 n = 8/136 [6%], BSEP3 n = 5/ 56 [9%]) died prior to LT (age 1.6 [1.1–3.5] years). |
| **Van Vaisberg, 2019[24]** | N = 11 (8 with PFIC)  PFIC1/2/5 (%): 62/12/25  Ileal exclusion | 1995 to 2018 | NR | 1 / 8 (12.5%) |
| **Wang, 2017[25]** | N = 58 (38 with PFIC)  FIC1/BSEP/GGTP<100 (%): 42/47/10  PEBD: 68%.  IE 15%: 13%  IE 40%: 3%  GBC: 16% | 2005 to 2013 | NR | 1 death, although unclear if ALGS/PFIC |

**Abbreviations:** ALGS, Alagille syndrome; BSEP, bile salt export pump; FIC1, familial intrahepatic cholestasis 1; GGTP, gamma-glutamyl transpeptidase; IE, ileal exclusion; LT, liver transplant; NR, not reported; PEBD, partial external biliary diversion; PILBD, Paucity of Interlobular Bile Duct; PFIC, progressive intrahepatic cholestasis; PFIC?: PFIC variant unknown; UDCA, ursodeoxycholic acid.
