# Supplementary material for: Epidemiology and burden of progressive familial intrahepatic cholestasis: a systematic review
Source: Orphanet J Rare Dis. 2021 Jun 3;16:255. doi: 10.1186/s13023-021-01884-4 (PMC8173883; doi:10.1186/s13023-021-01884-4)
Supplement: Supplementary file 3 — Additional file 3. Prevalence and mortality of PFIC in included studies. Results for research question 1. [file 13023_2021_1884_MOESM3_ESM.docx]

**Additional file 3**

**Prevalence and mortality of PFIC in included studies**

| **First author, year** | **Sample size**  **Type of PFIC**  **Relevant treatment** | **Study period** | **Prevalence** | **Mortality** |
| --- | --- | --- | --- | --- |
| **Acar, 2019[21]** | N= 3  PFIC3 (%): 100  Living-donor LT | Median posttransplant follow up, years (range): 3 (0.9-6) | NR | 0% |
| **Alhebbi, 2020[31]** | N = 193  PFIC 1/2/3/4 (%): 14/37/14/11  Treatment: NA | 1998 to 2020 | Of 1500 children referred, 455 were diagnosed with familial liver disorders.  193 were diagnosed with PFIC 1-4. | NR |
| **Bjornland, 2020[33]** | N=33  PFIC1/2/3 (%): 17/79/4  SBD | 1992 to 2018  Median follow-up time for total population: 10 (0.6–25.2) years | NR | Three deaths during follow-up. One due to complications after LT.  One due to adhesion ileus and sepsis following PBD. Third due to unknown cause. |
| **Flores, 2018[22]** | N = 37 (ALGS & PFIC)  PFIC1/2/3 (%): 11/24/11  14 patients LT; 2 internal diversion (PFIC); 1 external diversion (ALGS) | Jan 1996 to Dec 2016 | Patients admitted with PFIC from January 1996 to December 2016 confirmed by imaging, genetic or laboratory investigation: 17 | NR |
| **Malik, 2017[26]** | N = 644  Not differentiated  Treatment NR | Jan 2013 to Dec 2015 | Patients admitted 12/644 (1.87%)  8 males and 4 females | NR |
| **Meena, 2017[27]** | N = 632 biopsies  N/A  Treatment NR | NR | PFIC2 (n=14) and PFIC3 (n=1) most common cause of PILBD in infants.  In older children, PFIC was the largest etiological group (n = 6, 3 type-2, 3 type-3) | NR |
| **Morris, 2015[28]** | N = 6  Byler disease  Treatment NR | Jan 2007 to Oct 2014 | NR | 0 |
| **Ruth, 2018[30]** | N = 80  PFIC1/2/3 (%): 8/20/2  Unknown (%): 37  BRIC (%): 13  Treatment NR | 1984 to 2017 | NR | Number died:  PFIC1: 2  PFIC2: 2 |
| **Schatz, 2018[29]** | N= 38  PFIC3 (%): 100  24 patients received UDCA treatment.  Additional treatment of pruritus in 9 children: rifampicin and phenobarbital. | NR | NR | 2 children died following LT |
| **Thebaut, 2017[13]** | N = 20 (13 ALGS; 7 PFIC)  PFIC2/?: 43/56  Oral treatment with sertraline UDCA and rifampicin. | June 1, 2007 to May 31, 2014 | NR | Deaths: 1/20 (5%) |
| **Valamparampil, 2018[14]** | N = 25 with PFIC vs 50 controls  PFIC1/2/3/4 (%): 28/28/40/4  5 children had internal biliary diversion.  . | Median follow-up duration was 3.5 years (range 0.5 months - 6.5 years). | NR | 1 year graft and patient survival:84%. There was no late graft loss or mortality in this cohort. |
| **Valamparampil, 2019[23]** | N = 34  PFIC1/2/3/4 (%): 23/21/50/6  Patients received LT - 94% from a living donor | 2010 to 2018 | NR | Survival PFIC1: 63%  Survival PFIC2 or PFIC3 or PFIC4: 84.6%  BA: 91% |
| **Van Wessel, 2018[15]** | N = 203  BSEP-def: patients with ABCB11 mutations: mild (n=68), moderate (n=100) or severe (n=35)  UDCA: 47% at first visit | NR | NR | NR |
| **Van Wessel, 2018[16]** | N = 46  FIC1-def (%): 100  Use of UDCA prior to first visit 39% | NR | NR | Mortality prior to LT was 2%. |
| **Van Wessel, 2018[15]** | N = 226  FIC1-def (%): 19  BSEP-def (%): 81  Treatment NR | NR | NR | Pre-transplant mortality was 2% in FIC1-def and 5% in BSEP-def patients |
| **Van Wessel, 2018[16]** | N = 234  FIC1 def (%): 18  BSEP def (%): 82  Treatment NR | NR | NR | Pre-transplant mortality was 2% for FIC1-def and 4% for BSEP-def. |
| **Van Wessel, 2019[19]** | N = 55  PFIC1(%): 100  Treatment: NR | 3.2 (1.2-6.1) years | NR | Prior to LT 9% |
| **Van Wessel, 2020[20]** | N = 264  Patients with ABCB11 according to severity (BSEP1, BSEP2, BSEP3)  Treatment NR | Data collected by investigators within each centre since 1977. Data exported from REDCap on March 1, 2019 | NR | 16 patients (BSEP1 n = 3/72 [4%], BSEP2 n = 8/136 [6%], BSEP3 n = 5/ 56 [9%]) died prior to LT (age 1.6 [1.1–3.5] years). |
| **Van Vaisberg, 2019[24]** | N = 11 (8 with PFIC)  PFIC1/2/5 (%): 62/12/25  Ileal exclusion | 1995 to 2018 | NR | 1 / 8 (12.5%) |
| **Wang, 2017[25]** | N = 58 (38 with PFIC)  FIC1/BSEP/GGTP<100 (%): 42/47/10  PEBD: 68%.  IE 15%: 13%  IE 40%: 3%  GBC: 16% | 2005 to 2013 | NR | 1 death, although unclear if ALGS/PFIC |

**Abbreviations:** ALGS, Alagille syndrome; BSEP, bile salt export pump; FIC1, familial intrahepatic cholestasis 1; GGTP, gamma-glutamyl transpeptidase; IE, ileal exclusion; LT, liver transplant; NR, not reported; PEBD, partial external biliary diversion; PILBD, Paucity of Interlobular Bile Duct; PFIC, progressive intrahepatic cholestasis; PFIC?: PFIC variant unknown; UDCA, ursodeoxycholic acid.
