# Supplementary material for: Epidemiology and burden of progressive familial intrahepatic cholestasis: a systematic review
Source: Orphanet J Rare Dis. 2021 Jun 3;16:255. doi: 10.1186/s13023-021-01884-4 (PMC8173883; doi:10.1186/s13023-021-01884-4)
Supplement: Supplementary file 4 — Additional file 4. Studies reporting disease progression. Results for research question 1. [file 13023_2021_1884_MOESM4_ESM.docx]

**Additional file 4**

**Studies reporting disease progression**

| **First author, year** | **Sample size**  **Type of PFIC**  **Relevant treatment** | **Study period** | **Disease progression** |
| --- | --- | --- | --- |
| **Acar, 2019[21]** | N = 13  PFIC3 (%): 100  LT | Median follow up time: 3 years (range 0.9-6) | LT  Indications for liver transplantation were portal hypertensive bleeding, severe itching, growth failure and cirrhosis.  Four patients underwent BD prior to LT. |
| **Bjornland, 2020[33]** | N = 33  PFIC1/2/3 (%): 17/79/4  SBD | 1992 to 2018  Median follow-up time: 10 (0.6–25.2) years | Patients who underwent/ listed for LT  PFIC1: 50%  PFIC2: 11%  PFIC3: 100% |
| **Flores, 2018[22]** | N = 37  PFIC1/2/3 (%): 11/24/11  14 patients received LT; 2 internal diversion (PFIC); 1 external diversion (ALGS) | Jan 1996 to Dec 2016 | Of the 37 patients diagnosed with ALGS or PFIC, 17 underwent surgical intervention.  2 patients with PFIC received BD. 14 patients received LT, however the number of those with PFIC is unclear. |
| **Malik, 2017[26]** | N = 644  Not differentiated  NR | Jan 2013 to Dec 2015 | NR |
| **Meena, 2017[27]** | N = 632 biopsies  NA  Treatment NR | NR | 28 children listed for transplantation (biliary atresia 13, PFIC 6, PSC 2, Autoimmune-overlap 2, Langerhans cell histiocytosis 1 and others 4) with indications being portal hypertension, growth failure, decompensation, pruritus and cholangitis in 25, 19, 16, 4 and 2 children, respectively. |
| **Morris, 2015[28]** | N = 6  Byler disease  Treatment NR | Jan 2007 to Oct 2014 | Evidence of progressive liver disease not identified in this cohort.  At the last follow-up, none of the patients had splenomegaly and platelet counts ranged from 235 to 525 x 10^9^/L. |
| **Ruth, 2018[30]** | N = 80  PFIC1/2/3 (%): 8/20/2  Unknown (%): 37  BRIC (%): 13 | 1984 to 2017 | LT  PFIC1/2/3 (%): 6/7/NA  Unknown: NA  BRIC: NA |
| **Schatz, 2018[29]** | N = 38  PFIC3 (%): 100  UDCA, rifampicin, phenobarbital | NR | LT  16 of 26 children listed for LT 13 patients received LT  2 deaths due to LT-related complications.  Acute biopsy-proven rejection observed in 4 patients, and biliary complications occurred in 2 patients. |
| **Thebaut, 2017[13]** | N = 20 (13 ALGS; 7 PFIC)  PFIC2/? (%): 43/56  Sertraline UDCA and rifampicin  . | June 1, 2007 to May 31, 2014 | Two patients with PFIC underwent SBD which was closed because it was not efficient. |
| **Thompson, 2020[32]** | N = 19  BSEP def (%): 100  Maralixabat | >4.5 years | No ongoing patients were listed for liver transplant after >4.5 years of maralixibat. |
| **Valamparampil, 2018[14]** | N = 25 patients with PFIC vs 50 controls  PFIC1/2/3/4 (%): 28/28/40/4  IBD | The median follow-up: 3.5 years (range, 0.5 months - 6.5 years). | The median age at LT was 46 months (range 6-204 months) and the duration of hospitalization was 21 days.  Incidental HCC was noted in explants in 4 children (16%) which as significantly higher than BA explants. |
| **Valamparampil, 2019[23]** | N =34  PFIC1/2/3/4 (%): 23/21/50/6  LT | 2010 to 2018 | LT rejection  PFIC1: 2/8 (25%)  PFIC2 or PFIC3 or PFIC4: 6/26 (23%) |
| **Van Wessel, 2018[15]** | N = 203 BSEP-def: compound heterozygous or homozygous *ABCB11* mutations.  Mild: (n=68)  Moderate: (n=100)  Severe: (n=35).  UDCA | NR | SBD rates  5 years: 27%  10 years: 34%  Mild category patients were more likely to have undergone SBD (HR mild vs severe = 5.4, 95%CI 1.7-17.6; p<0.01).  Overall NLS  5 years: 63%  10 years: 46%  18 years: 34% (mild 51%, moderate 24%, severe 0%; p=0.004)  NLS significantly higher in SBD+ve than in SBD-ve patients (HR=0.44; 95%CI 0.23-0.88, p=0.007), independent of mild or moderate BSEP mutations.  HCC  Overall: 8% at median age 2.1 years (0.7 to 11.0)  Mild: 3%  Moderate:8%  Severe: 18%  p=0.04 |
| **Van Wessel, 2018[16]** | N = 46  FIC1-def: 100  UDCA | NR | SBD rates  5 years old: male 37%, female 50%  10 years old: male 43%, female 50%  p=0.15  NLS  5 years: Overall 74%, male 71%, female 88%  10 years: Overall 46%, male 49%, female 58%  p=0.87  5 years: Compound heterozygous: 78%, compound homozygous 72%  10 years: Compound heterozygous: 33%, compound homozygous 52%  p=0.61  18 years: 46%  No significant difference between SBD+ patients and SBD- patients [HR=1.16; 95% CI (0.33-4.01); p=.82].  HCC  Not encountered in any patient during follow-up. |
| **Van Wessel, 2018[15]** | N = 226  FIC1-def: 19  BSEP-def: 81  Treatment NR | NR | SBD rates  5 years: FIC1-def 33%, BSEP-def 28%  10 years: FIC1-def 39%, BSEP-def 35%  10 years BSEP: mild 57%, medium 21%, severe 14%; p<0.001  NLS  5 years: FIC1-def 73%, BSEP-def 61%  10 years: FIC1-def 51%, BSEP-def 46%  10 years BSEP: mild 60%, medium 37%, severe 32%; p<0.001)  FIC1-def: SBD+ vs. SBD- no significant difference  Combining medium and mild BSEP-def patients, SBD+ was associated with a significantly higher NLS, compared with SBD- [HR=0.37; 95%CI (0.24-0.68); P=.001].  Transplant  Before 5 years: FIC1-def 27%, BSEP-def 36%  Before 10 years: FIC-1 49%, BSEP-def 52%  HCC  FIC1-def: not seen  BSEP-def: 10% (mild 2%, medium 12%, severe, 21%)  p=0.006 |
| **Van Wessel, 2018[16]** | N = 234  FIC1-def: 18  BSEP-def: 82  Treatment NR | NR | SBD rates at five/ten years of age  5 years: FIC1-def 33%, BSEP-def 27%  10 years: FIC1-def 39%, BSEP-def 34%  NLS  5 years: FIC1-def 73%, BSEP-def 61%  10 years FIC1-def 51%, BSEP-def 45%  18 years: FIC1 51%, BSEP 32%  BSEP-def: SBD+ significantly higher than in SBD- [HR=0.37; 95%CI (0.18–0.74), p= 0.002]  FIC1-def: no significant difference between SBD+ and SBD- groups.  Transplant  Before 5 years: FIC1-def 27%, BSEP-def 37%  Before 10 years: FIC1-def 49%, BSEP-def 53%  HCC  FIC1-def: not observed  BSEP-def: 8% |
| **Van Wessel, 2019[19]** | N = 55  PFIC1 (%): 100  Treatment NR | 3.2 (1.2-6.1) years | SBD rates  15 years: female 85%, male 39%, p=0.04  NLS  18 years: 38%  SBD was not associated with NLS (HR=1.64; 95% CI 0.58-4.60; p=0.35).  Post-SBD SBA levels <100mmol/L had significantly higher NLS compared to 100mmol/L (5 years post SBD, 100% vs 30%; p=0.02) |
| **Van Wessel, 2020[20]** | N = 264  Patients with *ABCB11* categorized according to (BSEP1, BSEP2, BSEP3) genotypic severity  Treatment NR | Data were collected by investigators within each centre, who identified patients who had ever been under paediatric care (age 0–18 years) since 1977. Data were exported from REDCap on March 1, 2019 | Median NLS, years  BSEP1: 20.4  BSEP2: 7.0  BSEP3: 3.5  P<0.001  HCC  15 years: BSEP1 4%, BSEP2 7%, BSEP3 34%, p=0.001  SBD associated with significantly increased NLS (HR 0.50; 95% CI 0.27–0.94: p = 0.03) in BSEP1 and BSEP2.  SBA concentration <102 µmol/L or a decrease of at least 75%, shortly after SBD, reliably predicted NLS of ≥15 years following SBD (p <0.001) |
| **Van Vaisberg, 2019[24]** | N = 11 (8 with PFIC)  PFIC1/2/3 (%): 62/12/25  IE | 1995 to 2018 | Chronic liver disease: 6/8 (75%)  LT: 1/8 (12.5%)  ESLD + death: 1/8 (12.5%) |
| **Wang, 2017[25]** | N = 58 (38 with PFIC)  FIC1/BSEP/GGTP (%): 42/47/10  PEBD: 68%.  IE 15%: 13%  IE 40%: 3%  GBC: 16% | 2005 to 2013 | Indications for surgical diversion  Pruritus: 92%  Progression of liver disease: 59%  Two patients with GGTP < 100 who had undergone IE subsequently underwent revision to PEBD due to persistent severe pruritus.  GBC division: 3 FIC1, 3 BSEP, 0 GGTP<100  Three out of 16 FIC1 (2/12 following PEBD, 1/2 IE, 0/2 GBC) underwent transplant.  Six out of 18 BSEP (3/13 s/p PEBD, 3/3 s/p IE, 0/2 s/p GBC) underwent transplant. |

**Abbreviations:** BD, biliary diversion; BRIC, Benign recurrent intrahepatic cholestasis; BSEP, bile salt export pump; CI, confidence interval; ESLD, end-stage liver disease; GBC, gall bladder to colon diversion; GGTP, Gamma-glutamyl transpeptidase; HCC, hepatocellular carcinoma; HR, hazard ratio; IE, ileal exclusion; LT, liver transplant; NA, not applicable; NLS, native liver survival; NR, not reported; PEBD, partial external biliary diversion; PFIC, progressive intrahepatic cholestasis; PFIC?: PFIC variant unknown; SBA, serum bile acid; SBD, surgical biliary diversion; UDCA, Ursodeoxycholic acid.
