# Supplementary material for: Epidemiology and burden of progressive familial intrahepatic cholestasis: a systematic review
Source: Orphanet J Rare Dis. 2021 Jun 3;16:255. doi: 10.1186/s13023-021-01884-4 (PMC8173883; doi:10.1186/s13023-021-01884-4)
Supplement: Supplementary file 5 — Additional file 5. Summary of studies. Study characteristics for research question 2. [file 13023_2021_1884_MOESM5_ESM.docx]

**Additional study 5**

**Summary of studies**

| **First author, year** | **Sample size**  **Study design** | **Objective** | **Setting, location** | **Study period** | **Population** | **Relevant outcomes** |
| --- | --- | --- | --- | --- | --- | --- |
| **Acar, 2019[21]** | N = 13  Retrospective, medical records | Experience of LT in PFIC3 | Transplantation centre, Turkey | NR | Patients with PFIC3 | Surgery rate |
| **Agarwal, 2016[62]** | N = 24  Retrospective, medical records | To study the clinical and laboratory profile of children with PFIC and evaluate their outcome. | Institute of Liver and Biliary Sciences, India | Jan 2011 to July 2015 | Children and adolescents under 18 years of age, diagnosed as PFIC | Surgery rate |
| **Arnell, 2008[49]** | N = 13  Prospective, single arm | To describe 13 patients with PFIC who have undergone SBD. | Secondary care, Sweden | 1992 to 2005 | PFIC patients undergoing SBD | Relapse rate, surgery rate. |
| **Aydogdu, 2007[50]** | N = 12  Prospective, single arm | To analyse the demographic features, clinical and histopathological findings in patients who underwent liver transplantation for PFIC | Secondary care, Turkey | Sept 1997 to Oct 2006 | Patients with PFIC undergoing LT | Graft and patient survival |
| **Bull, 2018[59]** | N = 57  Retrospective, database | Outcomes of partial external biliary diversion, ileal exclusion, and liver transplantation in PFIC 1or BSEP deficiency | Setting unclear, USA/UK | Surgery between 1985 and 2004 | Patients with PFIC1 and 2 who had undergone one or more surgical procedures | Surgery rate, mortality |
| **Cantez, 2018[63]** | N = 15  Retrospective, chart analysis | To analyse the impact of different genetic mutations on clinical outcome in classical PFIC diseases | Tertiary care, Turkey | 2006 to 2017 | Children with genetically confirmed PFIC | Surgery rate |
| **Chen, 2018[64]** | N = 34  Retrospective, chart analysis | To evaluate the long-term efficacy of PEBD surgery for PFIC | Setting unclear, China | Aug 2003 to Nov 2014 | Clinically diagnosed PFIC | Surgery rate |
| **Dinler, 1999[55]** | N = 9  Prospective, single arm | To show the effect of UDCA on clinical, laboratory and histologic findings in children with Byler disease | Unclear setting, Turkey | 1 year | Children with Byler disease | Surgery rate and survival |
| **Emond, 1995[51]** | N = 17  Prospective, single arm | Evaluation of SBD as primary therapy in PFIC | Secondary care, USA | 1985 to 1991 | Patients with PFIC | Surgery rate |
| **Englert, 2007[39]** | N = 42  Retrospective, medical records | To report the results of patients treated medically, with PEBD, and LT. | Secondary care, Germany | NR | Children with PFIC | Surgery rate |
| **Erginel, 2018[65]** | N = 6  Retrospective, patient records | To evaluate six patients with PFIC who have undergone PIBD in long-term follow-up. | Setting unclear, Turkey | 2008 to 2010 | Diagnosis of PFIC | Surgery rate |
| **Foroutan, 2020[9]** | N = 44  Prospective, single arm | Long term follow-up of PFIC cases receiving PIBD | Hospital of Shiraz University of Medical Sciences. Iran | Nov 2010 to Oct 2018 | Patients with PFIC, whose pruritus was unresponsive to medication, who underwent PIBD, were included in this study. | Surgery rate |
| **Halaweish, 2010[60]** | N = 7  Retrospective, medical records | To describe long-term PFIC data | Secondary care, USA | 2004 to 2008 | PFIC patients undergoing SBD | Surgery rate |
| **Ismail, 1999[57]** | N =46  Retrospective, medical records | To evaluate experience with medical therapy as well as two types of surgical treatment used in children with PFIC. | Secondary care, Poland | 1979 to 1998 | Patients with PFIC | Surgery rate |
| **Jacquemin, 1997[52]** | N = 39  Prospective, two arms | To evaluate the clinical and biochemical effects of long-term oral UDCA administration in 39 children with PFIC | Secondary care, Belgium. | Mar 1998 to April 1995 | Children with PFIC | Surgery rate |
| **Jankowska, 2014[53]** | N = 56  Prospective, single arm | To analyse experiences with IE in children with PFIC | Secondary care, Poland | 1979 to 2010 | Children with PFIC | Surgery rate |
| **Nielsen, 2004[66]** | N = 46  Retrospective, records | Analysis of PFIC in indigenous families in Greenland | General population, Greenland | 1943 to 2003 | Families with PFIC1 | Survival |
| **Ruth, 2018[30]** | N = 80  Retrospective, medical records | To identify genotype/phenotype correlation with clinical course and medical/surgical intervention necessary | Liver Unit, UK | 1984 to 2017 | Patients with a genetic or phenotypic diagnosis of PFIC | Surgery rate |
| **Schatz, 2018[67]** | N = 38  Retrospective, medical records | To collect information on onset and progression of this entity in different age groups and to assess the relevance of this disease for the differential diagnosis of chronic liver disease | Hospital, Germany | NR | Patients with *ABCB4* gene | Surgery rate |
| **Schukfeh, 2012[58]** | N = 24  Retrospective, medical records | To assess the long-term outcome and complications after PEBD | Secondary care, Germany | 1994 to 2008 | All patients undergoing PEBD | Surgery rate |
| **Valamparampil, 2018[14]** | N = 25  Retrospective, no further details | Evaluate children with PFIC who underwent LT | NR, India | NR | Patients with PFIC | Survival |
| **Valamparampil, 2019[23]** | N = 51  Retrospective, medical records | To determine LT outcomes for paediatric recipients with PFIC as compared to BA. | Setting unclear, India | 2010 to 2018 | LT recipients with PFIC | Survival |
| **Van Wessel, 2018[15]** | N = 203  Retrospective, cohort study (NAPPED) | To better understand the natural course of PFIC2 and the efficacy of interventions | Global | NR | Patients with compound heterozygous or homozygous *ABCB11* mutations | Surgery rate  HCC |
| **Van Wessel, 2018[15]** | N = 226  Retrospective, cohort study (NAPPED) | To understand the nature of PFIC 1 and 2 and the efficacy of interventions. | Global | NR | FIC1-def and BSEP-def homozygous or compound heterozygous patients | Surgery rate |
| **Van Wessel, 2018[16]** | N = 234  Retrospective, cohort study (NAPPED) | To understand the nature of PFIC 1 and 2 and the efficacy of interventions | Global | NR | Patients who were compound heterozygous or homozygous for disease associated mutations in *ATP8B1* or *ABCB11* | Surgery rate |
| **Van Wessel, 2019[56]** | N = 51  Retrospective, cohort study (NAPPED) | To assess if biochemical parameters after SBD could function as a reliable surrogate parameter for long-term NLS. | Global | NR | Patients with compound heterozygous or homozygous pathological mutations in *ABCB11* with SBD. | Surgery rate |
| **Van Wessel, 2019[19]** | N = 55  Retrospective, cohort study (NAPPED) | To provide genotypical, phenotypical and biochemical factors associated with SBD and NLS in patients | Global | NR | Patients with compound heterozygous or homozygous disease associated *ATP8B1* mutations. | Surgery rate |
| **Varma, 2015[68]** | N = 22  Retrospective, patient records | To detect predictive markers for a successful outcome with non-transplant management and corresponding response characteristics | Unclear setting, Belgium | 1990 to 2014 | Children diagnosed with PFIC2. | Surgery rate, relapse rate |
| **Wanty, 2004[54]** | N = 49  Prospective, single arm | To review the experience of centre in terms of diagnosis, management, and outcome of 49 paediatric PFIC patients | Secondary care, Belgium | 15 years | Patients with PFIC | Transplant rate |
| **Wassman, 2018[47]** | N = 32  Prospective, comparative | To quantify the HRQL in patients with PFIC and to evaluate whether there is a difference in their HRQL depending on the surgical approach | Primary care, Germany | 1988 to 2010 | Patients diagnosed with PFIC | HRQL |
| **Yee, 2018[48]** | N = 68  Cross-sectional with survey | To evaluate perceptions of itch severity and HRQL among paediatric patients and caregivers before and after surgery | NR, UK/USA | NR | Adolescents (aged 12–17 years) and caregivers of children (<12 years) with PFIC | HRQL |

**Abbreviations:** BA, biliary artresia; BSEP, Bile salt export pump; HRQL, health-related quality of life; IE, Ileal exclusion; LT, liver transplant; NLS, native liver survival; NR, not reported; PBD, partial biliary diversion; PEBD, partial external biliary diversion; PFIC, progressive familial intrahepatic cholestasis; PIBD, partial internal biliary diversion; QoL, quality of life; SBD, surgical biliary diversion; UDCA, ursodeoxycholic acid.
