# Supplementary material for: Epidemiology and burden of progressive familial intrahepatic cholestasis: a systematic review
Source: Orphanet J Rare Dis. 2021 Jun 3;16:255. doi: 10.1186/s13023-021-01884-4 (PMC8173883; doi:10.1186/s13023-021-01884-4)
Supplement: Supplementary file 6 — Additional file 6. Summary of patient characteristics. Patient characteristics for research question 2. [file 13023_2021_1884_MOESM6_ESM.docx]

**Additional file 6**

**Summary of patient characteristics**

| **First author, year** | **Sample size**  **Reported population** | **Characteristics** | **Relevant treatment** |
| --- | --- | --- | --- |
| **Acar, 2019[21]** | N = 22  PFIC type 3 diagnosed by clinical, laboratory and genetic analysis | Median age, years (range): 2.4 (0.8-6)  Male (%): 77  PFIC3 (%): 100 | LT |
| **Agarwal, 2016[62]** | N = 24  Children and adolescents under 18 years of age diagnosed as PFIC | Median age of presentation, months: 5.5  Male (%): 58  PFIC1/2/3 (%): 8/79/13 | Medical management, PEBD, PIBD, LT |
| **Arnell, 2008[49]** | N = 13  Children who fulfilled the diagnostic criteria for PFIC and underwent PEBD from 1992 to 2005 were included in the study | Range of 6 months to 13 years  Male (%): 46  PFIC:NR | PEBD |
| **Aydogdu, 2007[50]** | N = 12  Children with PFIC diagnosed | Range of 3 to 108 months  Male (%): 50  PFIC: NR | LT |
| **Bull, 2018[59]** | N = 57 for PEBD  Criteria for inclusion were a clinical diagnosis of low-GGT PFIC and mutation(s) in  *ATP8B1* or *ABCB11*. | Median age at PEBD, years (IQR):  PFIC1, 1.6 (0.8-6.5); PFIC2 2.3 (1.4-4.8)  Male (%): PFIC1, 32; PFIC2, 60  PFIC1/2 (%): 37/60  Other (%): 3 | PEBD |
| **Cantez, 2018[63]** | N = 15  Children with genetically confirmed PFIC. Children with a clinical diagnosis of PFIC, but absent genetic analysis were excluded | Mean age, months (range): 2.9 (1.5-6)  Male (%): 54  PFIC1/2/3 (%): 7/67/27 | NA |
| **Chen, 2018[64]** | N = 34  Patients with PFIC undergoing PEBD for bile diversions in our institution. Diagnosis of PFIC was made by clinical criteria and liver biopsy | Median age at surgery, months (range): 19 (4–217 months)  Male (%): NR  PFIC1/2/3/low GGT: 32/38/15/15 | PEBD and LT |
| **Dinler, 1999[55]** | N = 9  Byler disease diagnosis | Mean age, years (SD): 4.6 (2.2)  Male (%): 33  PFIC1 (%): 100 | UDCA |
| **Emond, 1995[51]** | N = 17  Patients with PFIC | Mean age, years (SD)  SBD: 10.5 (7.1)  LT: 4.6 (3.4)  Male (%): NR  PFIC: NR | LT |
| **Englert, 2007[39]** | N = 42  Patients with PFIC treated medically, with PEBD and LT. | PFIC2:2 months – 10 years (3.2 years)  PFIC3: 2 months to 11 years (2.6 years)  Male (%): PFIC2, 46; PFIC3, 44  PFIC2/3 (%): 62/38 | PEBD, LT |
| **Erginel, 2018[65]** | N = 6  Diagnosis of PFIC made according to the existence of pruritus, early onset of cholestasis, typical biochemical, laboratory findings, and the exclusion of other cholestatic diseases. | Age range, years  Diagnosis: 0.5-2  Surgery: 2-5  Male (%): 67  PFIC1/2 (%): 100 | PIBD |
| **Foroutan, 2020[9]** | N = 44  Children with PFIC types 1 and 2, who underwent PIBD were included in this study. PFIC was diagnosed on base of clinical signs, laboratory findings, and liver histopathology. | Median age at operation (range): 29 months (2 months to 18 years)  Male (%): 57  PFIC 1 & 2 (%): 100 | PIBD following lack of response to UDCA/rifampin/cholestyramine and pheonbarbital |
| **Halaweish, 2010[60]** | N = 7  Patients diagnosed with PFIC who underwent SBD between 2004 and 2008. The diagnosis of PFIC was made according to established criteria: jaundice, cholestasis of early onset with pruritus, typical biochemical and histologic findings including appropriate immunostaining, molecular analysis, and exclusion of other causes of cholestasis. | NR  NR  PFIC1/2/3 (%): 71/14/14 | SBD |
| **Ismail, 1999[57]** | N = 46  All patients with PFIC treated in the institution between 1979 and 1998 | Age range: 10 months – 19 years  Male (%): 59  NR | UDCA, PEBD, LT |
| **Jacquemin, 1997[52]** | N = 39  Criteria for PFIC included: 1) a history of chronic cholestatic liver disease with hepatomegaly or hepatosplenomegaly for which all other known causes of childhood cholestasis were excluded; 2) normal extra- and intrahepatic bile ducts as judged by percutaneous transhepatic cholecystography; and 3) liver histology showing various degrees of portal and lobular fibrosis, and lobular cholestasis with or without ductular proliferation. | Mean age, months (SD; range) at time of diagnosis: 5 (7; 1-30)  Male (%): 51  NR | UDCA |
| **Jankowska, 2014[53]** | N = 9  PFIC provisional diagnosis was established based on anamnesis, clinical symptoms (jaundice, pruritus, and hepatomegaly), and laboratory findings (all children had low g-glutamyl transpeptidase activity and high concentrations of serum bile acids). | Median, years (range): 0.5 (0.3-13)  Male (%): 33  PFIC2 (%): 33  No mutation confirmed (%): 66 | IE |
| **Nielsen, 2004[66]** | N = 46  Detailed genealogy including clinical description and examination of families with PFIC | NR  NR  PFIC1 (%): 100 | NA |
| **Ruth, 2018[30]** | N = 80  All patients of BCH Liver Unit with a genetic or phenotypic diagnosis of PFIC presenting from 1984 - 2017. | Median age at diagnosis, days (range)  PFIC1: 213 (12-416)  PFIC2: 66 (9-1003)  PFIC3: 29 (29-47)  Unk: 49 (13-250)  BRIC: 5110 (40-10427)  Male (%): 46  PFIC1/2/3 (%): 10/25/3  Unk (%): 46  BRIC (%): 16 | SBD, LT, cholecystectomy |
| **Schatz, 2018[67]** | N = 38  Patient files and electronic health care records of 38 patients who presented with one of the clinical phenotypes (PFIC3, ICP, or LPAC syndrome) | Range of age:  5 months to 6 years 2 months  Male (%): 45  PFIC3 (%): 100 | UDCA, LT |
| **Schukfeh, 2012[58]** | N = 24  Patients with PFIC in this institution undergoing PEBD. PFIC was diagnosed according to established criteria: jaundice, early onset cholestasis with pruritus, typical biochemical and histologic findings and exclusion of other causes of cholestasis. | Median age at PEBD, months (range): 26 (4-17)  Male (%): 67  NR | PEBD |
| **Valamparampil, 2018[14]** | N = 25  Children with PFIC undergoing LT | NR  NR  PFIC1/2/3/4 (%): 28/28/40/4 | LT |
| **Valamparampil, 2019[23]** | N = 34 with PFIC  LT recipients with PFIC | NR  NR  PFIC1/2/3/4 (%): 24/21/50/6 | LT |
| **Van Wessel, 2018[15]** | N = 203  Patients with compound heterozygous or homozygous *ABCB11* mutations | Median age at first visit (range): 9 months (0-195)  Median age at last follow up (range): 5.5 years (0.1-31.4)  Male (%): 53  BSEP-def: patients with compound heterozygous or homozygous *ABCB11* mutations : mild (n=68), moderate ( n=100) or severe ( n=35). | UDCA: 47% at first visit |
| **Van Wessel, 2018[15]** | N = 226  Patients who were either homozygous or compound heterozygous for disease associated mutations in *ATP8B1* or *ABCB11* | Median age at first visit, months (range):  FIC1-def, 6 (0-201); BSEP-def, 9 (0-195)  Median age at last follow up, months (range):  FIC1-def, 69 (3-334); BSEP-def, 62 (1-487)  Male: NR  FIC1-def: 19; BSEP-def: 81 | NR |
| **Van Wessel, 2018[16]** | N = 234  Patients who were either homozygous or compound heterozygous for disease associated mutations in *ATP8B1* or *ABCB11* | Median age at first visit, months (range:)  FIC1-def, 6 (0-201); BSEP-def, 9 (0-195)  Median age at last follow up, months (range):  FIC1-def 69 (3-334); BSEP-def, 62 (1-487)  Male: NR  FIC1-def: 18; BSEP-def: 82 | NR |
| **Van Wessel, 2019[56]** | N = 51  Patients with compound heterozygous or homozygous pathological mutations in *ABCB11*, who had undergone SBD | Age: NR  Male: NR  BSEP-def: 100 | PEBD and IE, |
| **Van Wessel, 2019[19]** | N = 55  Patients with FIC1 deficiency | Median age at first visit, years (range): 0.5 (3-1.1)  Male (%): 77  FIC1-def: 100 | SBD |
| **Varma, 2015[68]** | N = 22  The diagnostic criteria for PFIC2 were taken as normal GGT cholestasis or neonatal hepatitis with absent canalicular BSEP on immunohistochemistry and/or presence of genetic changes in the *ABCB11* gene known to cause PFIC2 | Median age, months (range)  Responder: 9.5 (2-48)  Non-responders:3.17 (1-9)  Male: NR  PFIC2 (%): 100 | UDCA first-line, with PEBD considered as second-line.  LT according to nonresponse to UDCA or BD, HCC, or progressive disease. |
| **Wanty, 2004[54]** | N = 49  All patients met the published criteria of PFIC including history of chronic cholestatic liver disease with jaundice, hepatomegaly and/or pruritus | Median age, months (range)  PFIC 1 & 2: 2 (0-58)  PFIC 3: 3 (0-36)  Male (%): PFIC1 & 2: 60; PFIC3: 42  PFIC 1 & 2/3: 61/39 | UDCA, SBD, LT |
| **Wassman, 2018[47]** | N = 32  Patients who were treated with the diagnosis of a PFIC in their clinic between 1988 and 2010 | Mean age, years (SD): 17.7 (7.3)  Male (%): 47  NR | PEBD, LT |
| **Yee, 2018[48]** | N = 68  Adolescents (aged 12–17 years) and caregivers of children (<12 years) with PFIC | Mean age, years: 7.5 (2.3)  Male (%): 62  PFIC1/2/3 (%): 54/35/9  Unknown (%): 2 | BD, LT |

**Abbreviations:** ALGS, Alagille syndrome; BA, Biliary artresia; BSEP, bile salt export pump; FIC1, familial intrahepatic cholestasis 1; GBC, gallbladder to colon diversion; GGT, Gamma-glutamyl transferase; GGTP, gamma-glutamyl transpeptidase; IE, ileal exclusion; IQR, interquartile range; LPAC, low phospholipid-associated cholestasis; LT, liver transplant; ICP, intrahepatic cholestasis of pregnancy; LT, liver transplant; NA, not applicable; NR, not reported; PEBD, partial external biliary diversion; PFIC, progressive intrahepatic cholestasis; PIBD, Partial internal biliary diversion; SD, standard deviation; UDCA, Ursodeoxycholic acid.
