# Supplementary material for: Epidemiology and burden of progressive familial intrahepatic cholestasis: a systematic review
Source: Orphanet J Rare Dis. 2021 Jun 3;16:255. doi: 10.1186/s13023-021-01884-4 (PMC8173883; doi:10.1186/s13023-021-01884-4)
Supplement: Supplementary file 7 — Additional file 7. Results for question 2. [file 13023_2021_1884_MOESM7_ESM.docx]

**Additional file 7**

**Outcomes reported in included studies**

| **First author, year** | **Sample size**  **Type of PFIC**  **Relevant treatment** | **Study period** | **Burden of disease outcomes** |
| --- | --- | --- | --- |
| **Acar, 2019[21]** | N = 22  PFIC3 (%): 100  LT | NR  Median follow up: 3 years (range 0.9-6) | LT: 60%  Survival at 3 years: 100 |
| **Agarwal, 2016[62]** | N = 24  PFIC1/2/3(%): 8/79/13  Medical management, PEBD, PIBD, LT | Jan 2011 to July 2015 | PIBD: 17%  LT: 25% |
| **Arnell, 2008[49]** | N = 13  NR  PEBD | 1992 to 2005 | Post-PEBD  HCC: 8  LT: 8 |
| **Aydogdu, 2007[50]** | N = 12  NR  LT | Sept 1997 to Oct 2006 | 1-year graft survival: 69.2%  1-year patient survival:75% |
| **Bull, 2018[59]** | N = 145  PFIC1/2/other (%): 37/60/3  PEBD | 1985 and 2004 | PEBD: 39%  LT: 44%  IE: 4% |
| **Cantez, 2018[63]** | N = 15  PFIC1/2/3 (%): 7/67/27  Treatment: NA | 2006 to 2017 | 7 patients needed surgery: Five (4 PFIC2, 1 PFIC3) received PEBD (33.3%), three (2 PFIC2, 1 PFIC3) received LT (20%). One child with BD underwent LT. |
| **Chen, 2018[64]** | N = 34  PFIC1/2/3/Low GGT (%): 32/38/15/15  PEBD and LT | Aug 2003 to Nov 2014 | Two PFIC2 patients underwent LT. One patient with PFIC2 and one patient with PFIC3 died due to severe diarrhoea and dehydration; one PFIC2 patient died of intractable constipation.  Post-operative death: 1 |
| **Dinler, 1999[55]** | N = 9  PFIC1 (%): 100  UDCA | 1 year | Deaths: two patients died at 3 and 3.5 years of age.  One of them had undergone LT at 3.5 years old and died of transplantation complications within 1 month. |
| **Emond, 1995[51]** | N =17  NR  LT | 1985 to 1991  Mean follow-up period, 3.8 years; range, 2 to 6 years | SBD: 47  LT: 65  Of 11 LT, 8 were alive (1 to 5 years postoperatively).  The survival rate: 73% |
| **Englert, 2007[39]** | N = 42  PFIC2/3 (%): 62/38  PEBD, LT | NR | PEBD: 40 with 58% of these then undergoing LT.  LT: 55, with 5% on waiting list.  Patient survival of 100% and a primary graft survival of 89% |
| **Erginel, 2018[65]** | N = 6  PFIC1 & 2 (%): 100  PIBD | 2008 to 2010 | One patient died following LT |
| **Foroutan, 2020[9]** | N = 44  PFIC1/2 (%): 100  PIBD following lack of response to UDCA/rifampin/cholestyramine and pheonbarbital | Nov 2010 to Oct 2018 | Of 44 children, 14 were lost to follow up, 2 died, and 2 cases underwent LT. |
| **Halaweish, 2010[60]** | N = 7  PFIC1/2/3 (%): 71/14/14  SBD | 2004 to 2008 | Surgery revisions: 4 patients with persistent stoma-related complications requiring a total of 14 revisions.  LT: 29 (average, 44±18 months post-SBD)  Deaths: 29 |
| **Ismail, 1999[57]** | N = 46  NR  UDCA, PEBD, LT | 1979 to 1998 | PEBD:35  LT: 35 (including one after unsuccessful PEBD). |
| **Jacquemin, 1997[52]** | N = 39  NR  UDCA | Mar 1998 to April 1995 | SBD: 3  LT/waiting list: 23 |
| **Jankowska, 2014[53]** | N = 9  PFIC2 (%): 33  IE | 1979 to 2010 | PEBD: 11  LT: 11 |
| **Nielsen, 2004[66]** | N = 46  PFIC1 (%): 100  NA | 1943 to 2003 | 46 affected cases diagnosed since 1943. Six affected children are alive aged 1-21 years. |
| **Ruth, 2018[30]** | N = 80  PFIC1/2/3 (%): 8/20/2  Unknown: 37  BRIC: 13  NR | 1984 to 2017 | Patients with PFIC1 were more likely to require SBD or LT, 37.5% and 75%, respectively |
| **Schatz, 2018[67]** | N = 38  PFIC3 (%): 100  UDCA, rifampicin, phenobarbital | NR | NR  LT: 34  Deaths: 5 |
| **Schukfeh, 2012[58]** | N = 24  No differentiation  PEBD | 1994 to 2008 | LT: 38 |
| **Valamparampil, 2018[14]** | N = 25 patients with PFIC vs 50 controls  PFIC1/2/3/4 (%): 28/28/40/4  IBD | Median follow-up: 3.5 years (range, 0.5 month- 6.5 years). | 1-year graft and patient survival was 84%. No late graft loss or mortality in this cohort. |
| **Valamparampil, 2019[23]** | N = 34  PFIC1/2/3/4 (%): 23/21/50/6  LT | 2010 to 2018 | Survival PFIC1: 63%  Survival PFIC2 or PFIC3 or PFIC4: 84.6% |
| **Van Wessel, 2018[15]** | N = 203  BSEP-def: patients with *ABCB11* mutations: mild (n=68), moderate (n=100) or severe (n=35)  UDCA | NR | SBD rates  5 years: 27%  10 years: 34%  Mild category patients more likely to have SBD (HR mild vs severe = 5.4, 95%CI 1.7-17.6; p<0.01).  Overall NLS  5 years: 63%  10 years: 46%  18 years: 34% (mild 51%, moderate 24%, severe 0%; p=0.004)  NLS significantly higher in SBD+ve than in SBD-ve patients (HR=0.44; 95%CI 0.23-0.88, p=0.007), independent of mild or moderate BSEP mutations.  HCC  Overall: 8% at median age 2.1 years (0.7 to 11.0)  Mild: 3%  Moderate:8%  Severe: 18%  p=0.04 |
| **Van Wessel, 2018[15]** | N = 226  FIC1-def: 19  BSEP-def: 81  NR | NR | Pre-transplant mortality was 2% in FIC1-def and 5% in BSEP-def patients |
| **Van Wessel, 2018[16]** | N = 234  FIC1 def: 18  BSEP def: 82  NR | NR | Pre-transplant mortality was 2% for FIC1-def and 4% for BSEP-def. |
| **Van Wessel, 2019[56]** | N = 51  BSEP-def:100  SBD | NR | SBD was closed in 6 patients (mild = 2, moderate = 3, severe = 1) at 2.0 [0.1-4.0] yr after SBD, followed by LT in 5/6 patients at 6.3 [0.9-10.3]yr post-SBD.  LT: 32% of all patients, 2.7 [1.2-10.3] yr post-SBD |
| **Van Wessel, 2019[19]** | N = 55  PFIC1 (%): 100  NR | 3.2 (1.2-6.1) years | Mortality Prior to LT 9% |
| **Varma, 2015[68]** | N = 22  PFIC2 (%): 100  UDCA, SBD | 1990 - 2014 | SBD: 14  Patients were classified as responders or nonresponders on the basis of their response to non-LT treatment.  Of nine surviving responders, median relapse-free survival time was 72 months (95% confidence interval 48-96 months) and 5-year relapse-free survival was 75% (95% confidence interval 33-100%). |
| **Wanty, 2004[54]** | N = 49  PFIC1/2 (%): 61  PFIC3 (%): 39  UDCA, SBD, LT | 15 years | SBD: 10%  LT: 63%  Survival rate 5 years after LT: 92%  5-year survival rate of all children who underwent LT: 78% |
| **Wassman, 2018[47]** | N = 32  Not differentiated  PEBD, LT | 1988 to 2010 | PedsQL Scale: No significant difference in HRQL between patients with PFIC after LT and those after PEBD except for marginal difference in physical functioning/health (P = 0.07) and r a lower score in patient school functioning of patients after LTx (P = 0.01) (see Table 10.) |
| **Yee, 2018[48]** | N = 68  PFIC1/2/3 (%): 54/35/9  Unknown (%): 2  BD, LT | NR | Patients who underwent BD all experienced improvements in HRQL, mainly due to improved sleep (73.4%), improved mood (67.4%) and less itching (63.3%). |

**Abbreviations:** ALGS, Alagille syndrome; BD, biliary diversion; BRIC, benign recurrent intrahepatic cholestasis; BSEP, bile salt export pump; FIC1, familial intrahepatic cholestasis 1; GGTP, gamma-glutamyl transpeptidase; HCC, hepatic cell carcinoma; HR, hazard ratio; IE, ileal exclusion; LT, liver transplant; NA, not applicable; NLS, native liver survival; NR, not reported; PEBD, partial external biliary diversion; PFIC, progressive intrahepatic cholestasis; PIBD, partial internal biliary diversion; SBD, surgical biliary diversion; UDCA, ursodeoxycholic acid.
